# Vertically Integrated Electronics: New Opportunities from Emerging Materials and Devices

**DOI:** 10.1007/s40820-022-00942-1

**Published:** 2022-10-07

**Authors:** Seongjae Kim, Juhyung Seo, Junhwan Choi, Hocheon Yoo

**Affiliations:** 1 Department of Electronic Engineering, Gachon University, 1342 Seongnam-daero, Sujeong-gu, Seongnam, Gyeonggi-do 13120 Republic of Korea; 2 Center of Bio-Integrated Electronics, Northwestern University, Evanston, IL 60208 USA; 3 Querrey Simpson Institute for Bioelectronics, Northwestern University, Evanston, IL 60208 USA; 4 Present Address: Department of Chemical Engineering, Dankook University, 152 Jukjeon-ro, Suji-gu, Yongin, Gyeonggi-do 16890 Republic of Korea

**Keywords:** Vertical stacking, Three-dimensional integration, Metal routing, Via-hole, Two-dimensional semiconductors

## Abstract

Vertical three-dimensional (3D) integration is a highly attractive strategy to integrate a large number of transistor devices per unit area. This approach has emerged to accommodate the higher demand of data processing capability and to circumvent the scaling limitation. A huge number of research efforts have been attempted to demonstrate vertically stacked electronics in the last two decades. In this review, we revisit materials and devices for the vertically integrated electronics with an emphasis on the emerging semiconductor materials that can be processable by bottom-up fabrication methods, which are suitable for future flexible and wearable electronics. The vertically stacked integrated circuits are reviewed based on the semiconductor materials: organic semiconductors, carbon nanotubes, metal oxide semiconductors, and atomically thin two-dimensional materials including transition metal dichalcogenides. The features, device performance, and fabrication methods for 3D integration of the transistor based on each semiconductor are discussed. Moreover, we highlight recent advances that can be important milestones in the vertically integrated electronics including advanced integrated circuits, sensors, and display systems. There are remaining challenges to overcome; however, we believe that the vertical 3D integration based on emerging semiconductor materials and devices can be a promising strategy for future electronics.

## Highlights

- The vertically integrated electronic devices based on emerging semiconductor materials including organic, metal oxide, and two-dimensional materials are revisited.
- Comprehensive aspects of the device architecture, performance, and fabrication method of the vertically stacked electronics according to each semiconductor material are discussed.
- Recent advances in vertically integrated electronic devices for emerging applications such as advanced integrated circuits, sensors, and display systems are highlighted.

## Graphical abstract

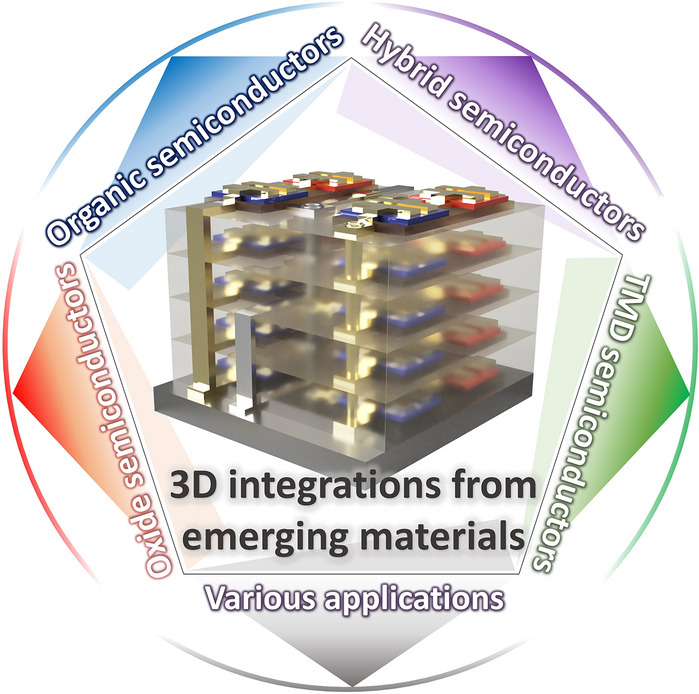

## Introduction

In 1959, Dawon Kahng and Mohamed M. Atalla first proposed metal oxide semiconductor field-effect transistors (MOSFETs), which leads to the success of silicon-based ICs as a key component in modern electronics. Approximately 13 sextillions (1.3 × 10^22^) of MOSFETs have been manufactured since it was presented in 1960 [1] and the MOSFETs have provided various applications such as not only processors [2, 3] but also image sensors [4, 5], memory integrations [6, 7], power electronics [8, 9], and neuromorphic systems [10, 11]. Dennard scaling suggests a transistor size is a key factor in determining its power consumption and operation frequency; thus, continuous efforts have been made to reduce the MOSFET dimension, which is the largest focus in the semiconductor societies and industries. However, the scaling reduction in the MOSFETs is encountering physical limitations. A feature size of a few nanometers on the level of a few atoms suffers from low process yield (~ 70%) and short channel effects.

**Fig. 1:**
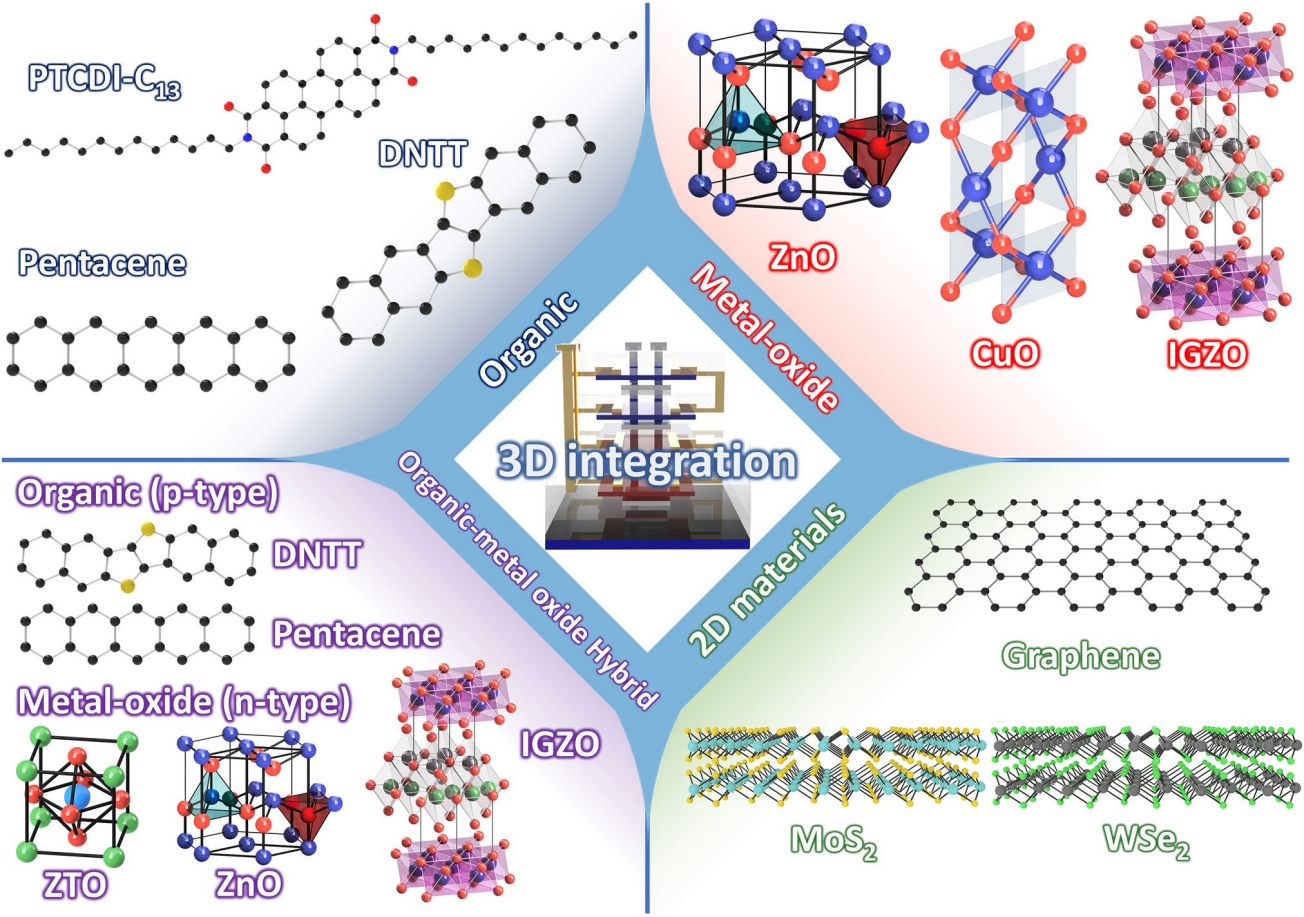
Overview of 3D integration based on emerging materials, including organic semiconductors, metal oxide semiconductors, and 2D materials

As an alternative approach, vertical integration has been considered a promising strategy to circumvent the issues in conventional silicon MOSFETs. Rather than top-down fabrication of silicon technologies, tremendous efforts on bottom-up process-based transistors and electronics have been made by adopting emerging semiconductor materials including transition metal dichalcogenides (TMDs) [12–14], graphene [15, 16], carbon nanotubes (CNTs) [17–19], organics [20–23], metal oxides [24, 25], and combinations of those materials [26, 27]. The largest difference from the conventional silicon MOSFETs is that each material can be simply deposited, which makes layer-by-layer vertical stacking available. This trait allows the devices to be vertically integrated without complex etching-based processes in top-down fabrication methods. There are considerable and increasing efforts to develop vertical integrations using the emerging semiconductor materials in the bottom-up approach, presenting promising feasibility of next-generation electronics. Furthermore, these emerging semiconductor materials offer additional advantages beyond the conventional MOSFETs. As a representative example of the additional properties, organic semiconductors provide a solution-processable fabrication [28–30], reducing the cost of electronic products, and two-dimensional (2D) TMDs are an atomically thin structure, reducing short channel effects [31] and less phonon scattering due to a van der Waals interface. Therefore, in such bottom-up-based devices, materials applications co-consideration is required as each material has strengths and weaknesses concerning device characteristics, fabrication process, and functional properties. Along this line, this review revisits recent progress in the emerging field of vertically integrated electronic devices and circuits enabled by the bottom-up process with emerging materials. With an emphasis on how the vertical stacking and integration can be made, this review summarizes representative examples depending on each material: organic, TMDs, CNTs, metal oxides, and hybrid combinations of such materials by organizing strengths/weaknesses and possibilities/challenges (Fig. 1). Furthermore, unique applications obtained by emerging materials-based vertical integrations are comprehensively reviewed, and through a timely overview, this review clarifies the benefits of the bottom-up process-based 3D integrations.

## Methods for Metal Interconnection

**Fig. 2:**
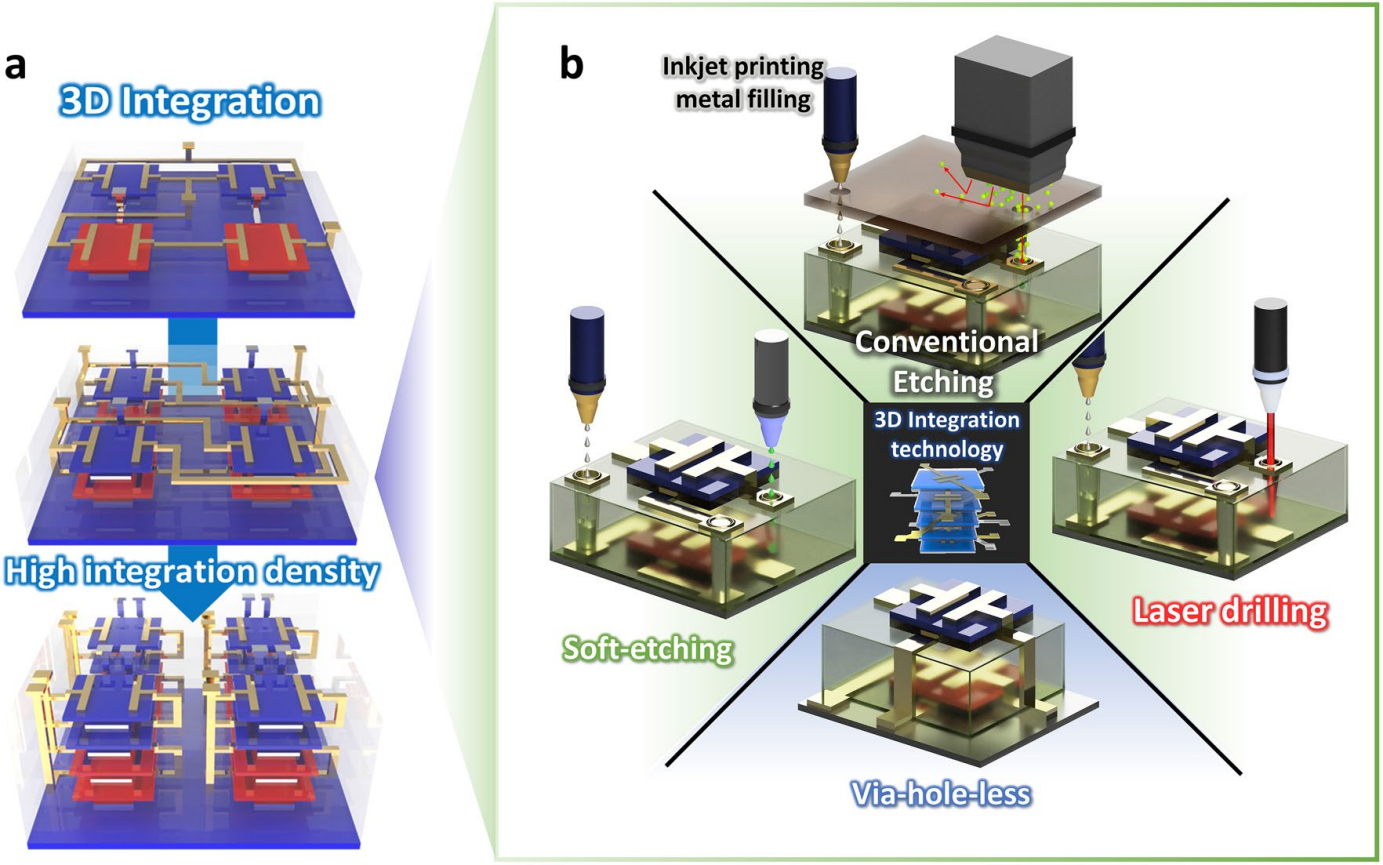
**a** Via-hole-based and **b** via-hole-less metal interconnection schemes for vertical integration to improve the integration density

Vertical 3D integration has emerged as a solution to overcome the scaling-based physical limitations and achieve high integration density within a given 2D planar area (Fig. 2a). To implement vertically stacked electronic devices, it is critically important to secure a reliable electrical connection between the electrodes on different layers. The metal interconnection methods can be divided mainly into the via-hole forming process and via-hole-less process (Fig. 2b). Conventional lithography-based patterning and etching are the representative methods for via-hole forming process. Most of metal oxide semiconductors and chemically robust 2D semiconductor materials are compatible with the lithography and wet/dry etching methods, and thus, via-hole forming methods based on etching have been widely used for those materials [25, 26, 32]. However, it is difficult to apply conventional lithography-based via-hole processes into 3D stacked organic devices because developers containing an organic solvent, plasma, or high-temperature process can damage the vulnerable semiconductors such as organic materials and thus can impair the device performance significantly [33–35]. For this reason, laser drilling or soft etching through solvent-based ink-jet printing has been utilized in organic electronic devices to make via-holes, by removing the dielectric layer in the selective area [36–38]. Nevertheless, such destructive methods may still have limitations. For example, irradiating high-energy laser is accompanied by the inevitable temperature rise, which can degrade the organic materials. In the solvent-based printing method, only dielectric materials that are soluble to the solvent can be used, which can limit the material selection. Alternatively, a via-hole-less multi-metal interconnection strategy was proposed by dielectric patterning [39]. A solvent-free deposition method for polymer dielectrics, called initiated chemical vapor deposition (iCVD), was utilized to achieve the robust insulating properties even with the ultrathin dielectric thickness [40–42]. Utilizing this all-dry method and shadow mask patterning, the polymer dielectric layer was directly patterned during the deposition process, which allows for the vertical interconnection without via-hole formation. The vertically stacked inverter circuits were fabricated by using transistors on 4 different floors verifying the reliable metal interconnection through this method [39]. Unlike planar structures, metal interconnections between different layers are critical for the vertically integrated devices. Therefore, a process design that is suitable for the materials constituting the device including semiconductor and dielectric materials is important.

## Vertically Integrated Electronic Devices Based on Emerging Semiconductor Materials

### Organic Materials-Based Vertical Integration

**Fig. 3:**
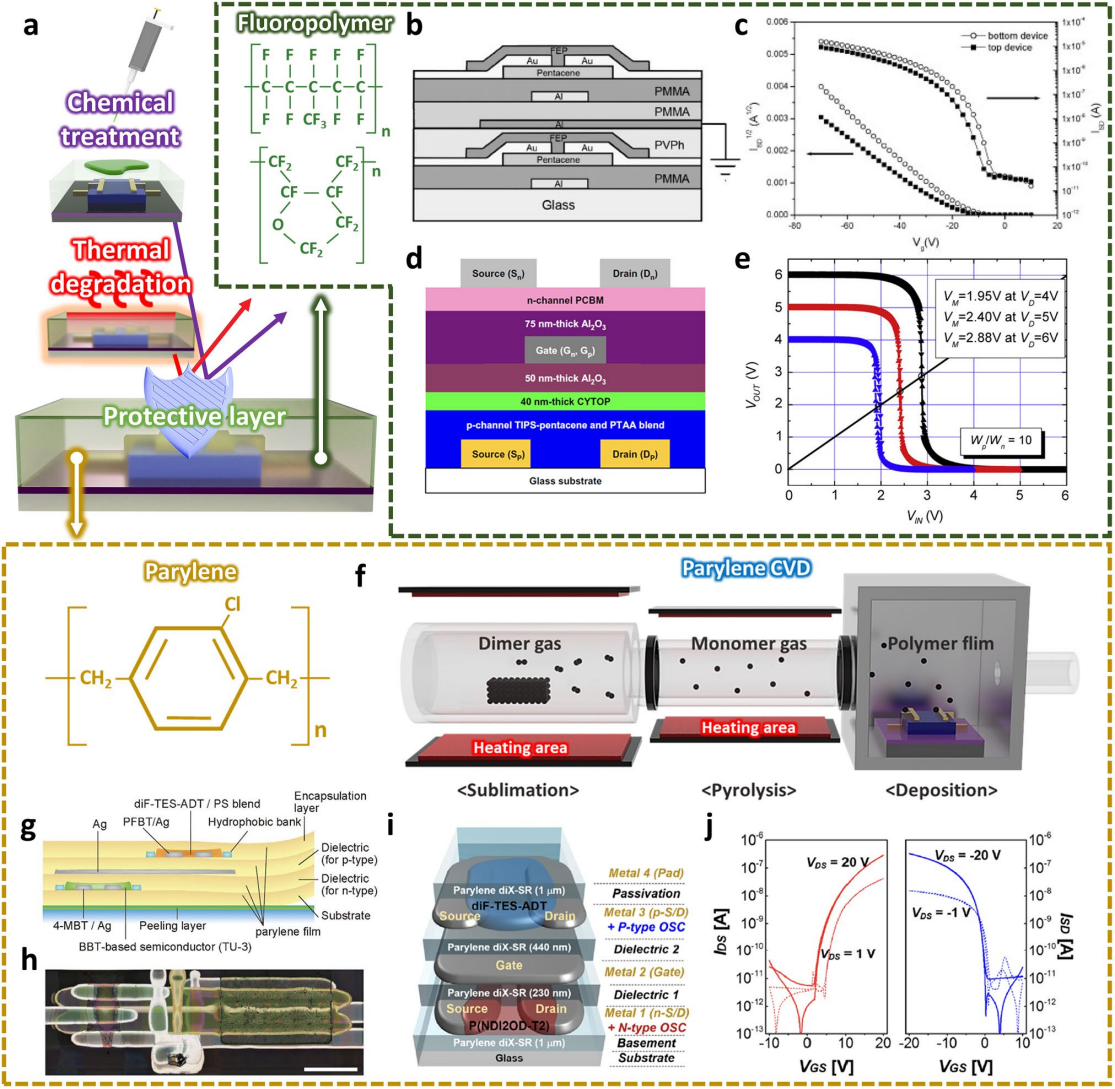
**a** Advantages obtained by using fluoropolymer or parylene as a protective layer in organic material-based vertical integration.** b** Schematic diagram realizing vertical stacking by applying FEP as a protective layer before forming an isolation layer.** c** Shift of the transfer curve by annealing after FEP deposition and PMMA coating [23]. Copyright © 2008, John Wiley and Sons.** d** Schematic diagram of a vertically stacked inverter with a structure that shares a gate composed of a blend semiconductor (TIPS-pentacene/PTAA) and PCBM with CYTOP as a protective layer. **e** Voltage transfer characteristics of the inverter [20]. Copyright © 2011, Elsevier.** f** Illustration of the process of deposition of parylene through the CVD method. **g, h** Schematic diagram and optical microscopy image of ultra-thin organic vertically stacked complementary inverter using parylene as gate dielectric and substrate [67]. Copyright © 2016, Springer Nature. **i** A schematic diagram of an organic vertical stacking inverter in which all processes except parylene used as the gate dielectric was implemented by ink jet printing. **j** The transfer curves of the P(NDI2OD-T2) OTFT and diF-TES-ADT/PS OTFT [71]. Copyright © 2016, American Chemical Society

Organic electronic devices have gained huge research attention for next-generation electronics due to their unique advantages such as low cost, large-area solution process suitability, intrinsic mechanical deformability, and light weight [43–46]. In addition, tunable electrical characteristics according to the molecular conjugated structures let them suitable for various electronic devices [47, 48]. With the growing interest in flexible electronics and human-friendly interfaces, demand for highly integrated electronic devices based on the organic thin-film transistor (OTFT) has been increasing. However, most of organic materials including organic semiconductors showed the limited thermal and chemical stability, which has been regarded as a critical obstacle in achieving 3D integration of the OTFTs (Fig. 3a). In particular, the solvent used in the following process can impair the electrical characteristics of the organic semiconductors [49, 50]. In addition, it is challenging to develop an organic semiconductor-based complementary circuit because n-type organic semiconductor materials are typically vulnerable to the ambient air [51, 52]. The degradation of electron transport in n-type organic semiconductors can occur due to electron trapping caused by the electrochemical reactions with water and oxygen. The organic semiconductors can be oxidized in the presence of water and oxygen in ambient air according to the following reaction [53, 54]:

1 $${\text{O}}_{2} + 2\,{\text{H}}_{2} {\text{O}} + 4\,{\text{osc}}^{ - } \rightleftarrows 4\,{\text{osc}} + 4\,{\text{OH}}^{ - }$$

This reaction in turn causes the transfer of electrons from the organic semiconductor to the OH– hydroxyl group, and thus, an OH– ion matrix immobilized in the channel is formed, at which electrons are trapped and not able to contribute transport.

Therefore, it has been important to protect the organic layers and devices to achieve the vertically stacked organic electronic devices. The organic device fabricated in the bottom or intermediate layers should be protected against organic solvents and other chemicals that are required in the subsequent process. In addition, thermal stress in device fabrication processes should be minimized. On the other hand, the organic devices in the topmost layer need to be protected from the ambient air. In this context, the polymer materials containing fluoroalkyl chain can be useful materials as well as maintaining the excellent interface with other organic materials. For example, Cytop has been widely utilized as a passivation material that can provide a strong hydrophobicity due to its low surface energy (~ 15 mJ m^−2^) [55]. This fluoropolymer can effectively protect the underlying device from water and oxygen and thus strongly supplement the environmental stability of n-type organic transistors [56]. Moreover, 3D integration can also be advantageous to improve the environmental stability of the OTFTs. By placing other layers on top of n-type OTFTs, potential degradation of the n-type organic semiconductor material caused by ambient air can be prevented. Moreover, in the 3D stacked structure, it is relatively easy to provide a proper interface and dielectric materials with different thicknesses for each organic semiconductor, by introducing different dielectric materials with different thicknesses, which can lead to the systematic optimization of the device performance of each OTFT independently. Therefore, high-density, organic integrated circuits have been developed by stacking OTFTs. In addition, to maximize the mechanical flexibility as well as OTFT performance, organic dielectric materials, mostly polymer materials, have been utilized in 3D organic electronic devices.

As discussed above, the damage to organic materials can be minimized by introducing fluoropolymers. Seo et al. [23] introduced fluoroethylenepropylene (FEP) that covered the pentacene semiconductor (Fig. 3b). This vapor-deposited fluoropolymer effectively protected the underlying OTFT, and the threshold voltage (*V*_th_) was also slightly decreased due to the thermal annealing effect (Fig. 3c). With the isolation layer, vertical integration of the OTFT was demonstrated. In the following study, by using different operation modes of OTFTs according to the dielectric materials, a unipolar inverter was demonstrated based on pentacene OTFTs [57]. The OTFT with poly(methyl methacrylate) (PMMA) dielectric layer was used as a driver transistor with enhancement mode. On the other hand, the OTFT with poly(vinyl phenol) (PVPh) dielectric layer could be operated with depletion mode, so it was utilized as a load transistor. The resulting inverter exhibited full swing in the voltage transfer curve (VTC), with high maximum DC gain (13.4 V/V) and noise margins (noise margin at the high level, NM_H_ = 47% and noise margin at the low level, NM_L_ = 33%).

CYTOP is another fluoropolymer that can prevent potential damage to the underlying organic semiconductors because fluorinated solvents for CYTOP are generally orthogonal to organic semiconductors [58, 59]. Combined with other advantages including excellent chemical stability and strong hydrophobicity, CYTOP has been used as a protective layer as well as a dielectric layer of top-gate geometry [60–63]. Kim et al. [20] utilized the bilayer dielectric where the CYTOP layer was spin coated and Al_2_O_3_ was deposited by atomic layer deposition (ALD) thereon (Fig. 3d). A top-gate, p-type OTFT based on 6,13-bis(triisopropylsilylethynyl) pentacene (TIPS-pentacene) and poly(triarylamine) (PTAA) blend semiconductor (TIPS-pentacene/PTAA) was fabricated by using the CYTOP/Al_2_O_3_ bilayer dielectric. A low-voltage operation of less than 7 V was achieved owing to the high dielectric constant of Al_2_O_3_, even though the metal oxide layer can limit the mechanical flexibility. A bottom-gate, n-type [6,6]-phenyl C_61_ butyric acid methyl ester (PCBM) OTFT was fabricated on top of the p-type OTFT with the shared gate electrode, leading to the low-voltage complementary inverter with high DC gain (24 V/V) (Fig. 3e). However, further optimization of the device geometry was required to improve the air stability, because ambient-instable n-type OTFT was positioned on the higher floor.

Parylene has been considered an attractive material for a dielectric layer as well as an ultrathin substrate because of its robust dielectric strength and excellent thermal and chemical stability [64–66]. In general, parylene can be fabricated by using the chemical vapor deposition (CVD) process, which makes it more attractive in the 3D stacked organic electronics (Fig. 3f). Takeda et al. [67] demonstrated the printed complementary inverter by utilizing parylene as a dielectric layer and substrate. The n-type benzobis (thiadiazole) (BBT) derivative (TU-3) OTFT was fabricated with a top-gate structure, and the p-type, bottom-gated 2,8-difluoro-5,11-bis(triethylsilylethynyl)anthradithiophene (diF-TES-ADT)/polystyrene (PS) OTFT was fabricated on top of the n-type OTFT by using a shared gate electrode (Fig. 3g,h). Those OTFTs were based on bottom-contact geometry, and the self-assembled monolayer (SAM) treatment was attempted on the source/drain (*S*/*D*) electrodes to improve the OTFT performance. The total thickness of the stacked device was less than 3 μm owing to the ultrathin parylene substrate (~ 1 μm), and it could be detached from the supporting glass substrate by introducing the peeling layer. Using this scheme, more complicated circuits were demonstrated including a 3-stage ring oscillator. The ring oscillator was affixed to the pre-stretched elastomer, and there was only a slight change in its force with the compressive strain as high as 20%. Another important advantage of the parylene CVD is its relatively low process temperature (~ 120 °C), which allows this process to be compatible with thermally vulnerable substrates.

An ink-jet printing process has gained huge attention for fabricating organic electronics due to its low-cost, large-area processability [68–70]. Also, a pattern can be defined during the printing procedure, which makes it more attractive to fabricate electronic devices. Kwon et al. [71] demonstrated 3D stacked organic integrated circuits by utilizing ink-jet printing. All the components consisting of the OTFT were fabricated via ink-jet printing, except for the parylene dielectric layer based on the CVD process. The p-type diF-TES-ADT/PS OTFT was vertically integrated with the bottom n-type poly{[N,N′-bis(2-octyldodecyl)naphthalene-1,4,5,8-bis(dicarboximide)-2,6-diyl]-alt-5,5′-(2,2′-bithiophene)} [P(NDI2OD-T2)] OTFT by sharing the shared gate electrode and this complementary OTFT structure (Fig. 3i,j), which was used as a building block for the integrated circuits. The ink-jet printed devices exhibited high yield with uniform electrical characteristics as well as long-term environmental stability. Based on the robust printing process and high uniformity therewith, the complex integrated circuits including the full adder were successfully demonstrated.

Typically, n-type organic semiconductors exhibit lower charge transport performance (i.e., carrier mobility) compared to p-type ones. In the 3D organic integrated circuits, matching the charge transport properties between p- and n-type OTFT can be achieved by varying/optimizing the dielectric materials and their thickness, which makes 3D stacking advantageous to achieve high-performance integrated circuits [72]. On the other hand, the electrical characteristics of the integrated circuits could also be optimized by implementing the dual-gate TFT structure, which allows for the precise *V*_th_ controllability as well as the improved device performance [73]. The ink-jet printable dithieno[2,3-d;2′,3′-d′]benzo[1,2-b;4,5-b′]dithiophene (DTBDT-C_6_) and TU-3 were used as p- and n-type organic semiconductor, respectively. A large-scale, printed logic circuit with 3D stacked structure was indeed fabricated on the flexible poly(ethylene naphthalate) (PEN) substrate, which exhibited high operational and environmental stability as well as low operating voltage (< 5 V). Those results showed the potential applicability of the printed organic integrated circuits to various computing system in flexible and wearable electronics.

### Metal Oxide-Based Vertical Integrations

Metal oxide semiconductors have been widely utilized in various research fields as well as display industries owing to their excellent electrical characteristics (i.e., high mobility) and intrinsic transparency [74–76]. Due to the tremendous research efforts in process optimization, the process temperature has been continuously reduced, which resulted in the reduction in thermal budget and demonstration of metal oxide semiconductors-based 3D integrated devices. Various n-type oxide semiconductor materials have been discovered including zinc oxide (ZnO) [77, 78], indium(III) oxide (In_2_O_3_) [79, 80], and indium gallium zinc oxide (IGZO) [81, 82]. These various n-type metal oxide semiconductors typically exhibit the excellent electron mobility, originated from oxygen vacancies [83]. However, it has been studied that the movement of hole carriers is relatively limited compared to that of electrons because the valence band of metal oxide semiconductor comprises hybrid orbitals of *p* and *d* orbitals [84]. As a result, it has been challenging to develop high-performance p-type metal oxide semiconductors. Nevertheless, with the great research efforts, the charge transport characteristics of p-type metal oxide semiconductors such as copper (II) oxide (CuO) [85, 86], and tin (II) oxide (SnO) [87, 88] have been improved, which expands their applicability into metal oxide semiconductor-based complementary inverters and logic circuits.

**Fig. 4:**
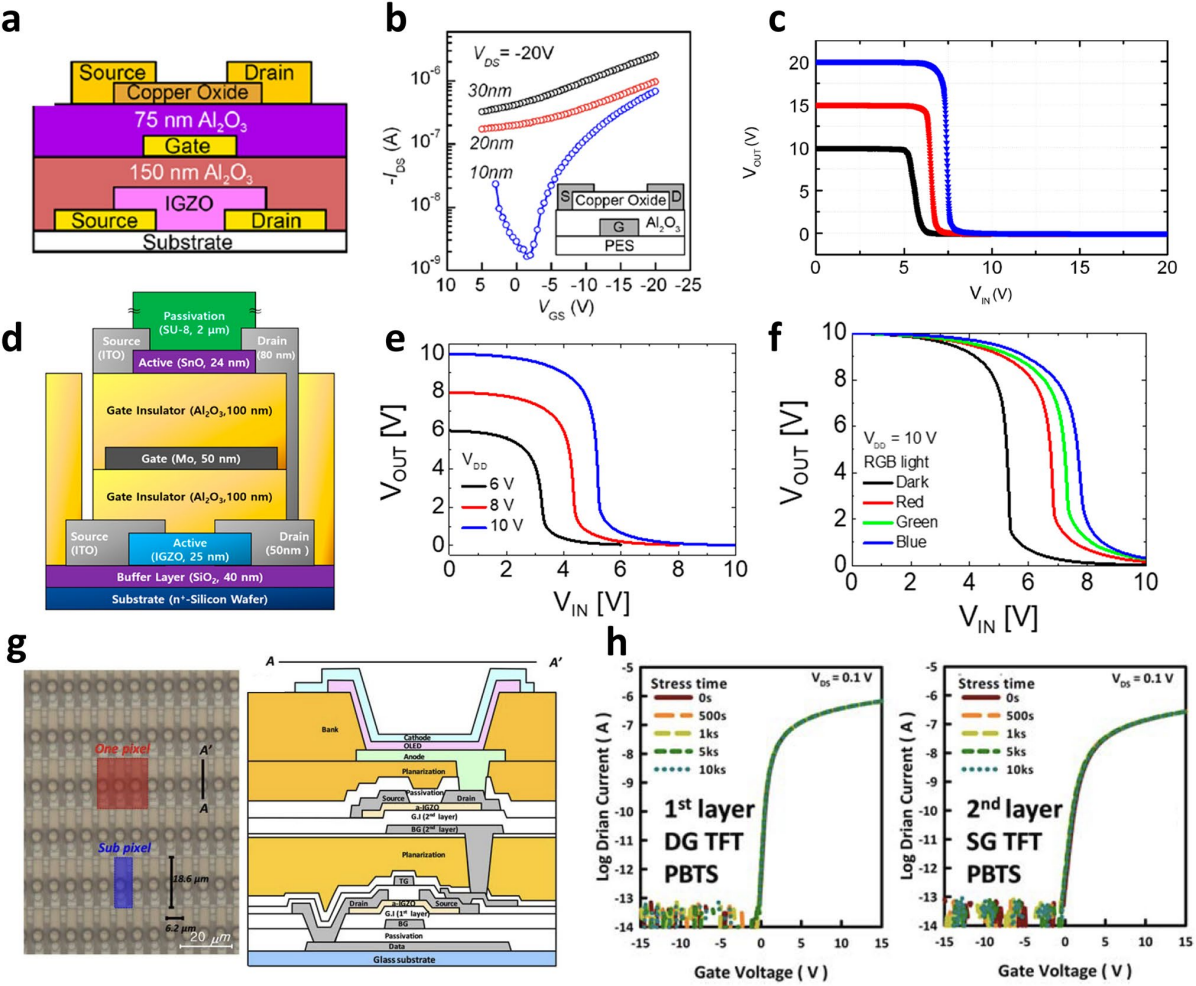
**a** Schematic diagram of a complementary inverter in which a p-type copper oxide transistor is vertically stacked on top of an n-type *α*-IGZO transistor.** b** Transfer curve of CuO TFT when CuO thickness is 10, 20, and 30 nm.** c** Voltage transfer characteristics of a vertically stacked inverter composed of a copper oxide transistor and an α-IGZO transistor [24]. Copyright © 2011, AIP Publishing. **d** A schematic of vertically stacked complementary inverter composed of a p-type SnO and an n-type IGZO TFTs.** e** Voltage transfer characteristics of a complementary inverter in which n-type IGZO TFT and p-type SnO TFT are vertically stacked when *V*_DD_ is 6, 8, and 10 V. **f** Change in the inverter characteristics according to red, green, and blue light application [25]. Copyright © 2019, MDPI. **g** Optical image and cross-sectional schematic of vertically stacked metal oxide TFT arrays for high-resolution active-matrix organic light-emitting diode backplanes.** h** PBTS measurement results for switching and driving TFTs, which are the first and second TFT layers for the backplane realization of high-resolution TFTs [89]. Copyright © 2020, John Wiley and Sons

Dindar et al. [24] fabricated a complementary inverter with a shared gate structure in which p-type CuO TFT and n-type IGZO TFT were vertically integrated (Fig. 4a). They optimized the electrical characteristics of the CuO TFTs according to the thickness of the CuO (Fig. 4b). When the thickness of CuO was above 20 nm, CuO was highly conductive and the off-state could not be secured in CuO TFT. On the other hand, when the CuO thickness was reduced to 10 nm, the current on/off ratio (*I*_on_/*I*_off_) was secured up to 3.9 × 10^2^ due to the improved off-state, which is sufficient to operate as a p-type TFT. Based on the improved p-type TFT, they demonstrated the vertically integrated inverter based on the metal oxide semiconductors, with a maximum gain as high as 120 V/V (Fig. 4c). The CuO TFT and IGZO TFT showed relatively unbalanced noise margins due to the large on-current difference. It was also noted that the device performance can be further improved by optimizing the channel geometry of the two TFTs and the thickness of the gate dielectric. Joo et al. [25] implemented the vertically integrated inverter using SnO, another p-type metal oxide semiconductor, along with IGZO TFT. A shared gate structure was utilized to achieve the inverter, and the interconnection between the drain electrodes of the top and bottom TFTs was made through via-holes formed by etching (Fig. 4d). They designed the channel width/length ratio of SnO TFT to be 7 times larger to compensate for the relatively low carrier mobility of the SnO TFT compared to that of the IGZO TFT. Thereby, the balanced inverter characteristic was achieved and the DC gain of the inverter reached 33.6 V/V with the supply voltage (*V*_DD_) of 10 V (Fig. 4e). They investigated how the characteristics of the inverter are modulated with respect to wavelength and intensity of light, to utilize the vertically integrated inverter as an optical sensor. With the light irradiation, the *V*_th_ of the SnO TFT located on the top layer shifted into positive direction, which induced positive shift in the VTC of the vertically integrated inverter. The amount of shift in VTC became larger as the wavelength of light decreased and the intensity increased (Fig. 4f). Through this achievement, it can be noted that the functionality per unit area can also be improved through 3D integration of the logic and optical sensor devices. The 3D integration can also be attractive when applied to a display device. Lee et al. [89] demonstrated a two-layered IGZO TFTs backplane for driving a high-resolution display (Fig. 4g). The IGZO TFTs in the 1st and 2nd layers were utilized as switching and driving TFTs, respectively, and N_2_O plasma was applied to secure the stability and reliability of the IGZO TFT. As a result, stable TFT characteristics were achieved even in the positive bias temperature stress (PBTS) under *V*_GS_ = 20 V and 60 °C for 10,000 s (Fig. 4h). The basic circuit of the OLED display is composed of the TFT that derived the OLED and a switching TFT that transmits voltage data. Through these vertically stacked structures and the data line placed under the switching TFT, they were able to reduce the pixel size by 83%, compared to the standard structures, which realized high resolution display. In addition, a dual gate structure was introduced, which led lower subthreshold swing (0.14 V dec^−1^) compared to those obtained in the single (bottom or top) gate structure.

### Organic–Metal Oxide Hybrid Combinations for Vertical Integrations

**Fig. 5:**
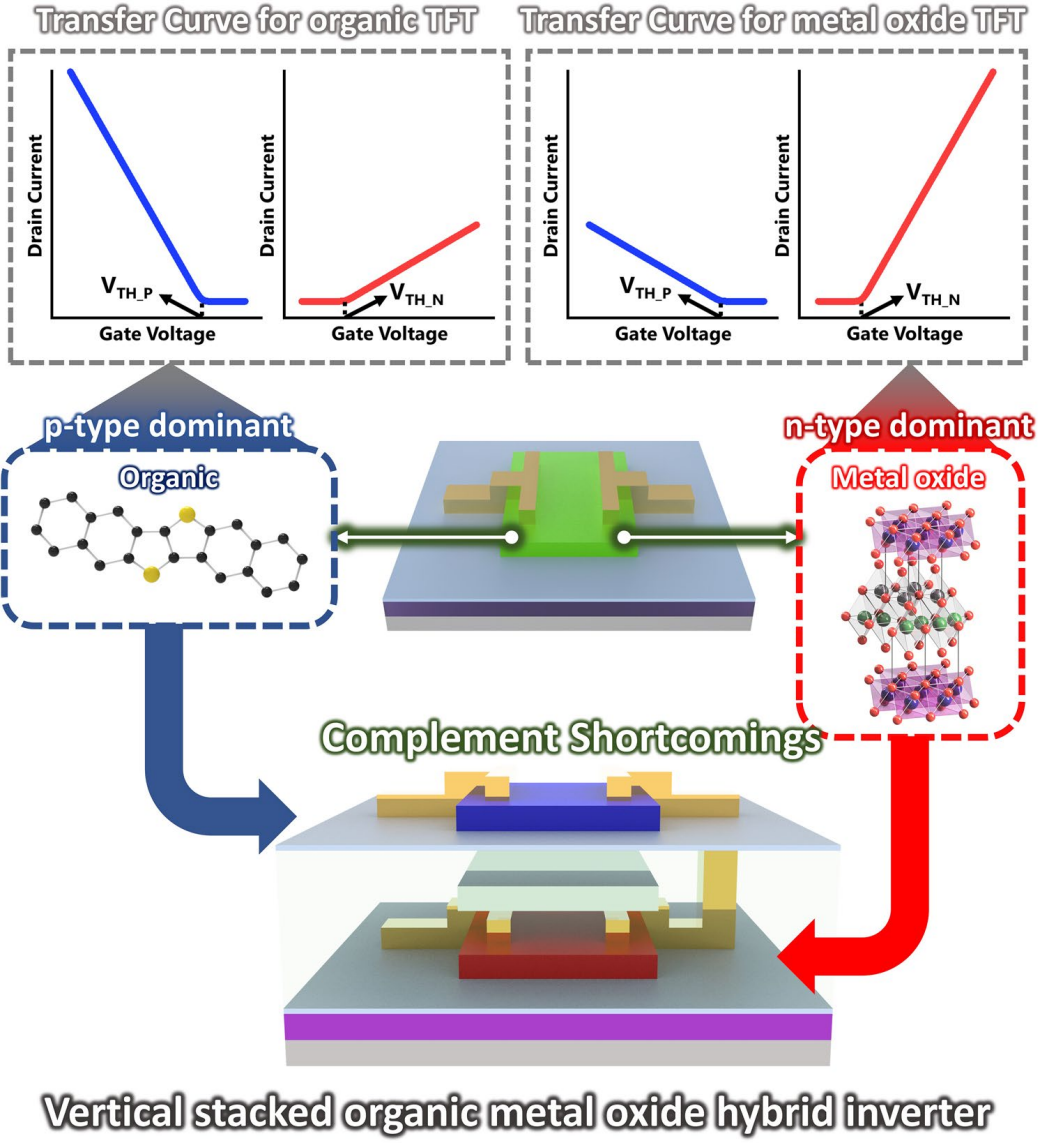
Schematic diagram of a vertical stacked p-type organic semiconductor and n-type metal oxide hybrid inverter capable of complementary operation

The hole mobility in metal oxide semiconductors is relatively limited compared to the electron mobility, as we mentioned above. On the other hand, the charge transport characteristics, as well as environmental stability, are superior in p-type semiconductors compared to n-type ones in organic materials. Therefore, the 3D integration of TFTs utilizing p-type organic semiconductors and n-type metal oxide semiconductors is an attractive way to overcome the shortcomings of each material (Fig. 5).

**Fig. 6:**
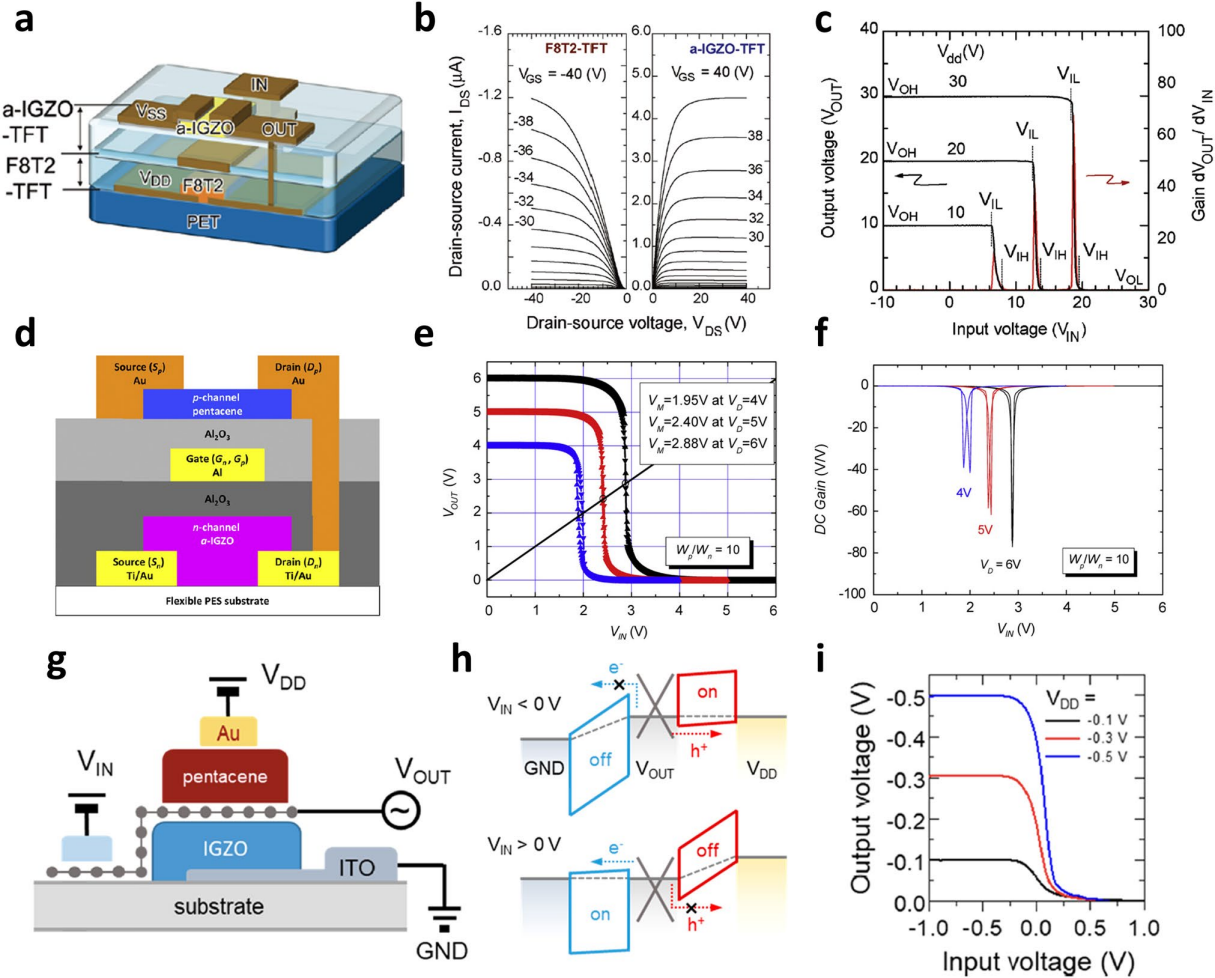
**a** Schematic diagram of a vertically stacked inverter with a structure in which the F8T2 TFT and the IGZO TFT share a gate electrode.** b** Output curves of the F8T2 TFT and IGZO TFT.** c** Voltage transfer characteristics of the vertically stacked organic-metal oxide hybrid inverter [90]. Copyright © 2010, AIP Publishing. **d** Schematic diagram of the vertically stacked organic-metal oxide hybrid inverter composed of IGZO TFT and pentacene TFT. **e, f** Voltage transfer characteristics and DC gain profiles of IGZO-pentacene vertically stacked inverters at *V*_DD_ of 4, 5, and 6 V [91]. Copyright © 2011, Elsevier.** g** Schematic device structure of a vertically stacked complementary inverter based on vertical Schottky barrier transistors composed of pentacene and IGZO. **h, i** Realization of inverter characteristics by controlling the Schottky barrier of junction between pentacene and graphene, IGZO and graphene through Fermi level modulation of graphene [15]. Copyright © 2019, American Chemical Society

Nomura et al. [90] fabricated the complementary inverter by vertically integrating p-type poly-(9,9-dioctylfluorene-co-bithiophene) (F8T2) OTFT and n-type IGZO TFT (Fig. 6a). The vertically stacked inverter could be fabricated on a flexible polyethylene terephthalate (PET) substrate because all the manufacturing processes could be carried out at temperature below 120 °C. They utilized the bottom-contact structure of the OTFT to prevent potential thermal damage to the organic semiconductor because the highest process temperature was required for the *S*/*D* electrode pattern of the F8T2 OTFT. Parylene was employed not only as of the gate insulator of IGZO TFT but also as the protection layer for F8T2 OTFT. Both F8T2 OTFT and IGZO TFT showed a low off-current of about 10^–13^ A and an *I*_on_/*I*_off_ of over 10^7^. Also, each output curve showed a clear current saturation (Fig. 6b). The resulting vertically stacked inverter showed full swings from *V*_DD_ to ground (*G*_ND_) (Fig. 6c). However, due to the relatively low dielectric constant of the gate insulator for the F8T2 OTFT and IGZO TFT (3.6 and 2.8, respectively), the operating voltage was relatively high (~ 30 V). Therefore, introducing a high-*k* dielectric was highly required to lower the driving voltage of the vertically stacked devices. Kim et al. [91] demonstrated a vertically stacked inverter capable of low-voltage operation by utilizing Al_2_O_3_ as a gate dielectric layer. The IGZO TFT and pentacene OTFT were fabricated on the bottom and top layers, respectively (Fig. 6d). The pentacene OTFT was placed on the top layer to prevent damage to the organic semiconductor in forming Al_2_O_3_ in the ALD process. They designed the channel width/length ratio of pentacene OTFT to be 10 times that of IGZO TFT, and the thickness of the gate insulator was independently controlled for the pentacene OTFT and IGZO TFT to achieve the balanced electrical characteristics between pentacene OTFT and IGZO TFT. As a result, the resulting inverter showed the switching voltage formed at the half of *V*_DD_, high DC gain (= 61 V/V) as well as low operating voltage (Fig. 6e,f). More complex logic circuits have been demonstrated based on vertical integration, by utilizing organic and oxide semiconductors. Kudo et al. [26] implemented vertically stacked inverter, NAND, and NOR circuits using the solution-processed TIPS-pentacene OTFT and ZnO TFT. They used silicone resin as a gate insulator and interconnected each electrode through via-holes formed by photolithography for NAND or NOR circuit implementation. Most of the existing vertically stacked inverters employed a shared gate structure and required a connection between the upper and lower layers of drain electrodes. On the other hand, Choi et al. [15] demonstrated the vertically integrated inverter without complex interconnection, by using graphene. Figure 6g shows a schematic diagram of the vertically stacked inverter based on the proposed vertical Schottky barrier transistors. The Schottky barrier formed at the junction between graphene and each semiconductor (pentacene and IGZO) was indirectly controlled by ion-gel dielectric and a gate electrode that was positioned laterally away from the graphene/semiconductor heterojunction. As a result, a full swing inverter characteristic was realized by controlling the Schottky barrier between pentacene and IGZO through the Fermi level modulation of graphene according to the gate voltage (Fig. 6h,i).

### 2D Materials-Based Vertical Integration

With the unique and excellent electrical and optoelectronic properties as the thickness reduces to atomic scale, 2D semiconductors have emerged as next-generation electronic materials. In particular, TMD materials including molybdenum disulfide (MoS_2_) and tungsten diselenide (WSe_2_) showed the excellent charge transport characteristics as well as tunable bandgap according to the number of layers in the 2D structure [92]. In addition to these advantages, 3D integration has been actively studied due to their unique heat dissipation mechanism and improved density due to atomic scale thickness. In the vertically stacked structure, it becomes difficult for the upper layer to dissipate the heat generated during operation with the increasing integration density. Therefore, thermal conductivity is an important factor to consider in the 3D integration. It has been studied that the atomically thin thickness of 2D materials can significantly reduce the thickness of each layer and the thickness of the insulating film between layers, thereby minimizing total dielectric thermal resistance and self-healing. Furthermore, the great potential of 2D materials has been reported, in that, it is possible to improve the density by more than 10 times compared to the conventional TSV-based 3D integration and 2.5 times compared to the conventional monolithic 3D integration [93, 94]. The following introduces the footprints of several researchers to realize 3D integration of the 2D material-based devices.

**Fig. 7:**
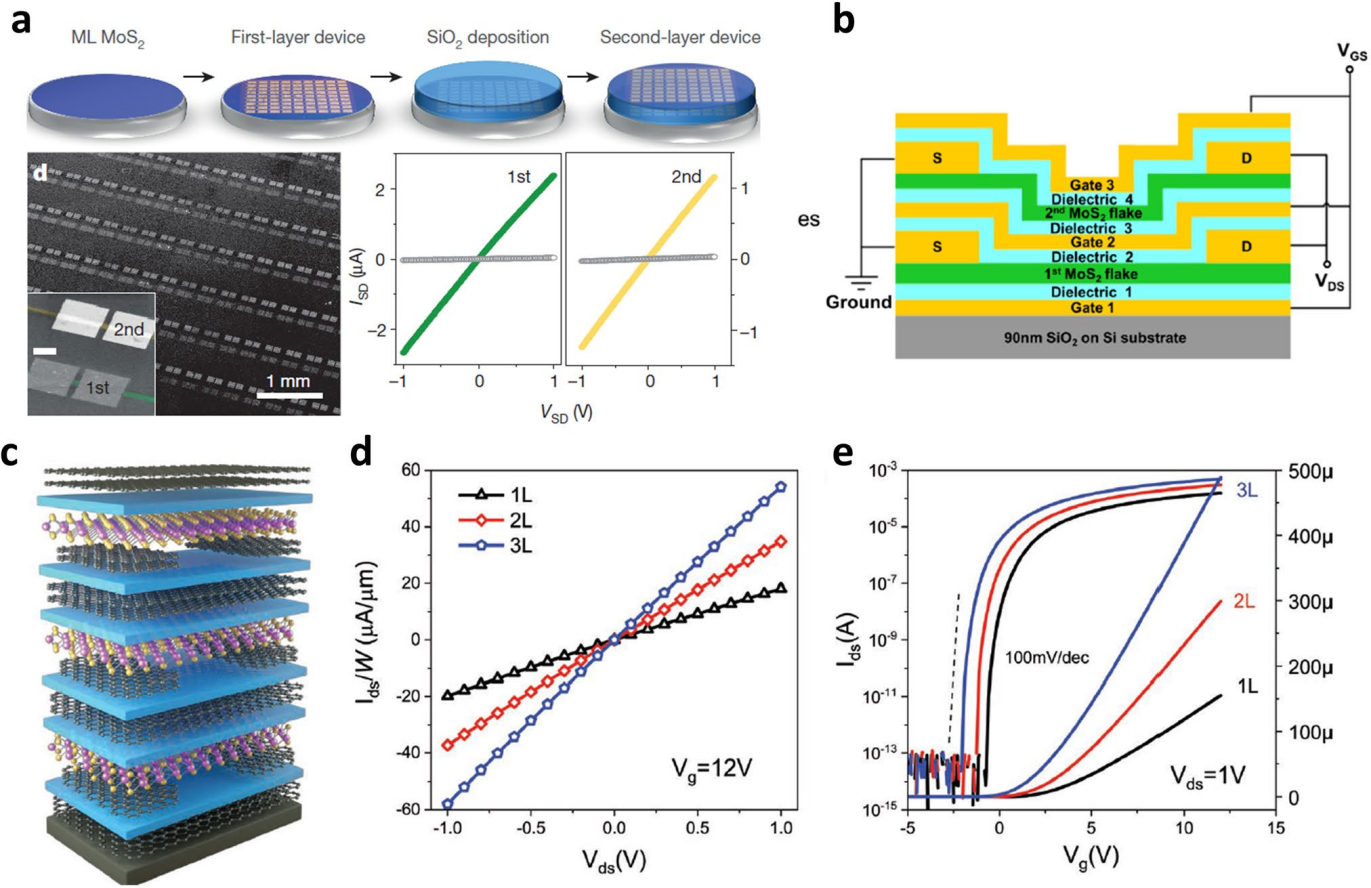
**a** Two-layer MoS_2_ TFTs manufacturing process through MOCVD process and optical microscope image of fabricated device and output curve characteristics of MoS_2_ TFT located on each layer [95]. Copyright © 2015, Springer Nature.** b** Schematic diagram of vertically stacked multi-channel MoS_2_ FET structure to improve current driving capability through effective channel length reduction [96]. Copyright © 2018, IEEE.** c** Illustration of a vertically stacked structure of three MoS_2_ TFTs, all layers of which are composed of 2D materials. **d, e** Current in output curve and transfer curve increasing with the number of vertically integrated MoS_2_ channels [13]. Copyright © 2020, John Wiley and Sons

In the early stage, mechanical exfoliation is a useful method to discover and investigate the electrical characteristics of 2D materials. However, large-area synthesis is eventually required not only to secure the practical use with high reproducibility but to demonstrate complex, vertically stacked devices. Kang et al. [95] grew highly uniform monolayer MoS_2_ and tungsten disulfide (WS_2_) on a large-area substrate with a yield of over 99% using the metal–organic chemical vapor deposition (MOCVD) process. Highly uniform and excellent electrical characteristics including mobility independent of the channel length of the transistor were demonstrated. They fabricated MoS_2_ channels in three different layers by repeatedly depositing SiO_2_ and MoS_2_ based on the optimized MOCVD process, which led to the first demonstration of the large-area, 3D integration of the 2D material-based TFTs (Fig. 7a). However, due to the global back gate operation, the drain current level of the MoS_2_ TFT fabricated in the upper layer was reduced compared to the MoS_2_ TFT in the first layer, which can cause the increasing operating voltage with the increasing number of layers. This problem can be solved by forming the gate and the gate dielectric on each TFT device. Zhou and Appenzeller [96] stacked two MoS_2_ TFTs vertically, to increase the effective channel width while maintaining the device area. Then, the gate electrodes of the two MoS_2_ TFTs were connected to each other, and the drain and source electrodes were also configured identically to demonstrate high current driving capability (Fig. 7b). Furthermore, Tang et al. [13] demonstrated 3D integration of three MoS_2_ TFTs where all the components consisted of 2D materials, including the channel material as well as the gate, gate dielectric, and *S*/*D* electrode (Fig. 7c). By connecting electrodes with the same function, the effective channel width was improved while maintaining the device area so that the current level of the MoS_2_ TFT increased in proportion to the number of devices (Fig. 7d,e). Despite simple stacking of the MoS_2_ TFTs, these researches directly showed the advantage of the vertical integration, where high current driving capability can be achieved by improving an effective channel width within the given area.

**Fig. 8:**
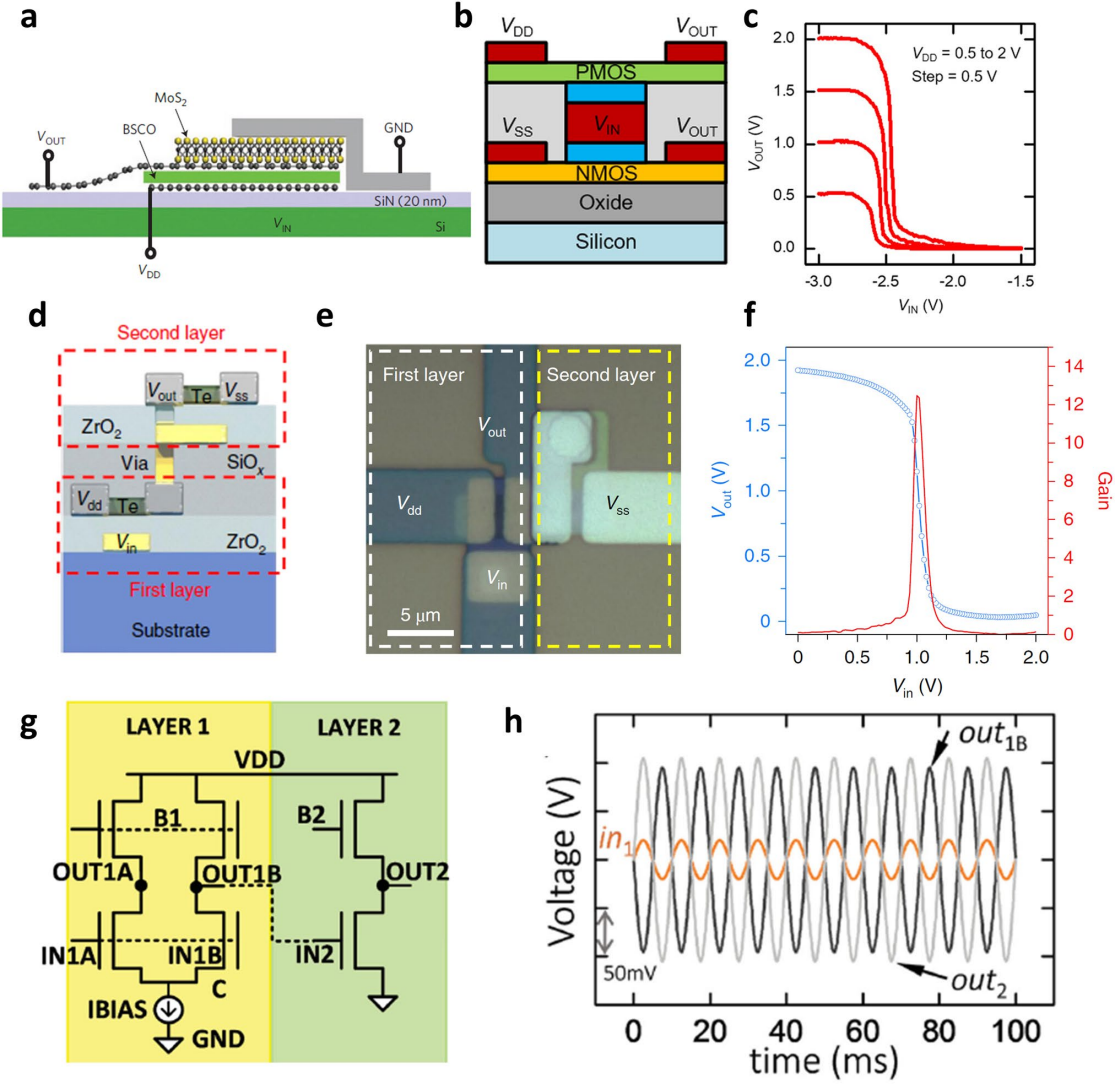
**a** Illustration of a vertically stacked inverter based on vertical transistors composed of MoS_2_ and Bi_2_Sr_2_Co_2_O_8_ [97]. Copyright © 2013, Springer Nature. **b** A schematic diagram of a vertically stacked inverter with a structure in which n-type MOS_2_ TFT and p-type WSe_2_ TFT share a gate. **c** Voltage transfer characteristics of the vertically stacked inverter [12]. Copyright © 2017, AIP Publishing. **d, e** Schematic and optical microscopy images of the thermal deposited Te TFT-based vertically stacked inverter structures. **f** Voltage transfer characteristics of Te-based vertically stacked inverter [32]. Copyright © 2020, Springer Nature.** g** Circuit diagram of differential amplifier (bottom layer) and common source amplifier (top layer) designed using MoS_2_ and WSe_2_. **h** Output of differential amplifier (bottom layer) and common source amplifier (top layer) circuit for an input signal with a peak-to-peak voltage of 50 mV [98]. Copyright © 2016, John Wiley and Sons

Yu et al. [97] demonstrated a complementary inverter by vertically integrating graphene, Bi_2_Sr_2_Co_2_O_8_, and MoS_2_ as shown in Fig. 8a. The vertically integrated inverter could be driven with this structure because the electric field of the bottom gate penetrated the p-type Bi_2_Sr_2_Co_2_O_8_ device and modulated the n-type MoS_2_ channel due to the weak screening effect of graphene. Consequently, a vertically integrated inverter was demonstrated and logic can be implemented in this relatively simple structure. On the other hand, as in the vertically integrated organic and metal oxide semiconductor devices, a shared gate structure has been utilized in the 3D integration of 2D material-based TFTs. Sachid et al. [12] demonstrated a complementary inverter using n-type MoS_2_ TFT and p-type WSe_2_ TFT by sharing the gate electrode as shown in Fig. 8b. They employed a high-*k* ZrO_2_ gate dielectric layer that led to low operating voltage (~ 3 V) of the TFTs and resulting vertically stacked inverter (Fig. 8c). The inverter exhibited full swing from *V*_DD_ to *G*_ND_ with a maximum voltage gain as high as 45 V/V.

As we mentioned above, it is important to secure large-area uniformity of 2D materials for reliable and reproducible device fabrication. Most large-area synthesis of 2D TMDs has relied on MOCVD, and a high process temperature is required to ensure the excellent charge transport characteristics. Zhao et al. [32] fabricated wafer-scale tellurium (Te) TFTs through simple thermal evaporation. Moreover, the process temperature was optimized to be as low as − 80 °C, which is fully compatible with plastic substrates. The fabricated Te TFT had hole mobility of 25–35 cm^2^ V^−1^ s^−1^, regardless of the substrate. By using the Te TFTs, they demonstrated several digital circuits such as lateral inverter, NAND, full adder, and 2-bit multiplier with high uniformity. In addition, the vertically integrated inverter was implemented by interconnecting Te TFTs on the top and bottom layers where one Te TFT was used as an active load and the other Te TFT acted as a driver, which proved the possibility of 3D integration of Te TFT (Fig. 8d,e). The fabricated 3D inverter showed DC gain characteristics of 12 V/V, and the operating voltage was as low as 2 V owing to the use of the high-*k* ZrO_2_ dielectric (Fig. 8f). In addition to the digital circuits, research efforts have been performed to implement functional analog circuits through the 3D integration of 2D semiconductor material-based TFTs. Sachid et al. [98] implemented analog circuits including the differential amplifier, common source amplifier, and signal mixer as well as digital circuits, by using MoS_2_ and WSe_2_ TFTs. Figure 8g shows the circuit diagram of the implemented differential amplifier (bottom layer) and common-source amplifier (top layer). The differential amplifier in the bottom layer was operated in single-ended operation mode. An output signal of a peak-to-peak voltage of about 270 mV and a voltage gain of 5.4 V/V was obtained with the applied peak-to-peak voltage of 50 mV as an input signal (Fig. 8h).

**Fig. 9:**
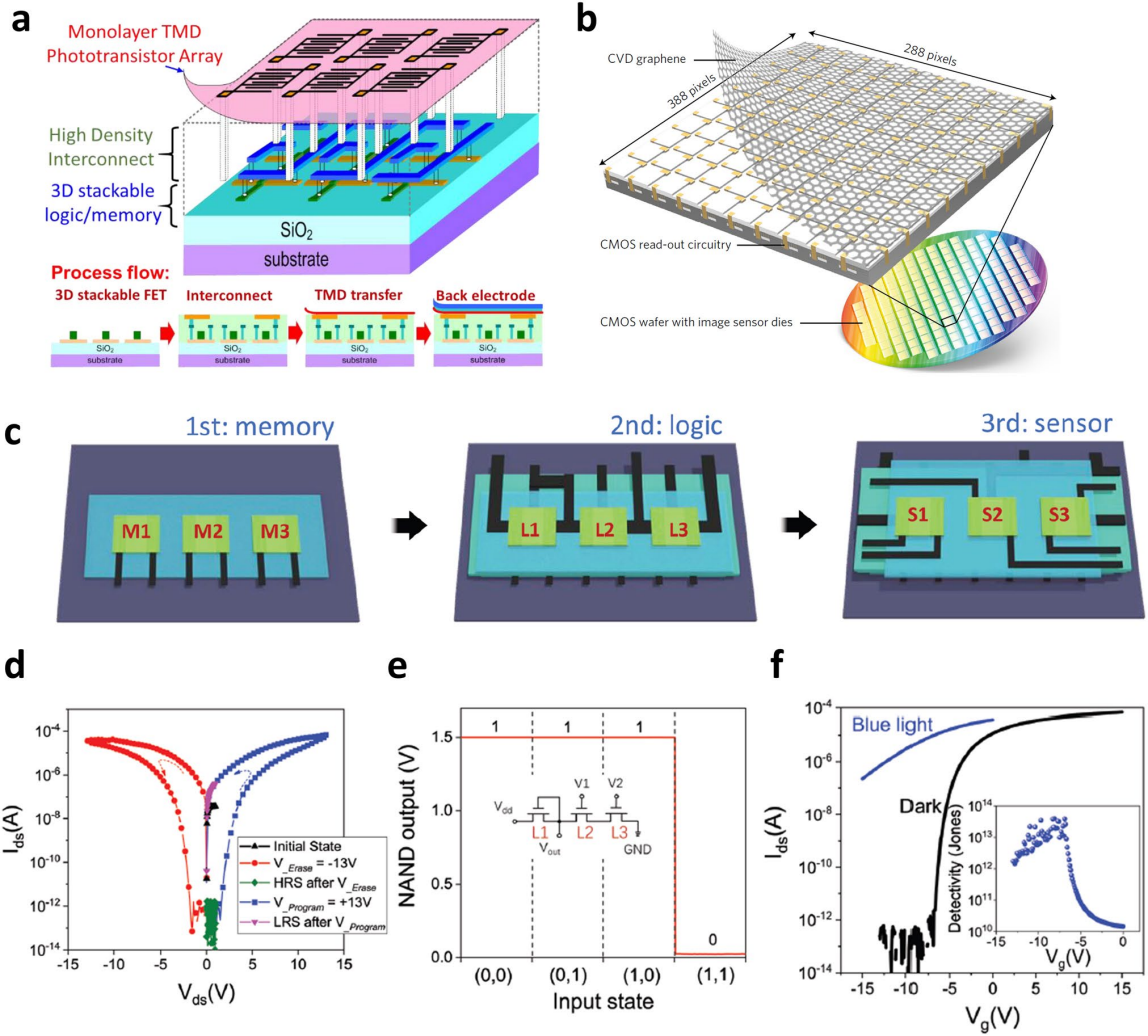
**a** Schematic diagram of a monolithic 3D image sensor with a monolayer TMD phototransistor array integrated on Si nanowire FET-based logic/memory hybrid 3D integrated circuits [99]. Copyright © 2016, IEEE. **b** Back-end-of-line CMOS integration of CVD graphene with 388 × 288 pixel image sensor read-out circuit [16]. Copyright © 2017, John Wiley and Sons.** c** Schematic diagram in which various functions such as memory, logic, and optical sensor based on 2D materials are vertically stacked on different layers. **d-f** Independent operation characteristics of memory (1st layer), logic (2nd layer), and optical sensor (3rd layer) located in each layer [13]. Copyright © 2020, John Wiley and Sons

As discussed above, integrated circuits by vertically integrating 2D material-based TFTs have been widely investigated. On the other hand, other kinds of devices such as sensors and memory can be vertically integrated to implement specific functions, as we discussed in the vertically integrated devices based on organic and metal oxide semiconductors. An important example in 2D semiconductor materials can be optoelectronic devices such as photodetectors and phototransistors, as TMDs exhibit excellent photoresponsivity with monolayer thickness. Yang et al. [99] implemented a phototransistor by transferring CVD-synthesized monolayer MoS_2_ onto the Si nanowire FET-based logic/memory hybrid 3D integrated circuits (Fig. 9a). Moreover, the sensing range can be controlled in this structure by using another monolayer TMDs including WS_2_, WSe_2_, and MoSe_2_ with a different band gap in addition to monolayer MoS_2_. In the photodetector, it is important to generate current by absorbing light without recombination of the photogenerated hole and electron in the channel. Therefore, the use of high conductivity materials such as graphene can be a great option to separate excess photogenerated carriers and effectively generate high photocurrent. Goossens et al. [16] demonstrated a high-performance image sensor array through 3D integration of complementary circuits and graphene (Fig. 9b). The graphene layer was inserted between the lead sulfide quantum dots (PbS QDs) photoactive layer and the CMOS read-out circuit. Through this structure, photogenerated holes and electrons from PbS QDs are transported to graphene. The optical sensing signal could be detected by the change in the conductivity of graphene, which showed significantly improved results compared to the devices without graphene. These studies highlighted the advantages of optoelectronic devices developed by vertical integration of the devices based on 2D materials including TMDs and graphene. In addition to the excellent semiconducting properties of TMDs, 2D materials can also exhibit high electrical conductivity (graphene) and insulating properties [hexagonal boron nitride (hBN)]. Exploiting these electrical properties of 2D materials, Tang et al. [13] demonstrated all 2D materials-based electronics, by utilizing MoS_2_, hBN, and graphene as semiconducting, insulating, and contact/gating materials, respectively. They also manufactured the vertically stacked electronic device, where different functional devices based on 2D materials including memory, logic (inverter, NAND), and sensor (optical sensor) were stacked (Fig. 9c). Figure 9d–f shows the electrical characteristics of each device based on the independent operation. Furthermore, they demonstrated cooperative operation between different devices by interconnecting the memory device (1st layer) and the optical sensor (3rd layer) to show the change of the memory state according to the photoresponse of the optical sensor. Despite the relatively small-scale fabrication, it is still meaningful that all layers composed of the devices were implemented by 2D materials. The improved integration density and large-area processing technology are still required in 2D material-based electronics. However, considering the excellent electrical characteristics of 2D materials and the extensive researches on 2D materials, we believe significant progress in vertical integration of 2D material-based devices should be achieved in near future.

### CNTs-Based Vertical Integration

CNTs have been spotlighted because they have advantages such as high electrical conductivity, thermal conductivity, and mechanical strength even with light weight. In addition, the electrical characteristics of carbon nanotube field effect transistors (CNTFETs) can be modulated into p-type, n-type, and ambipolar charge transport, through passivation [100, 101]. These characteristics make it possible to implement a complementary logic device only with CNTFETs.

Kanhaiya et al. implemented a vertically stacked complementary inverter by only using CNTFETs [17]. To implement a complementary inverter, p-type and n-type transistors are required, respectively. However, it was confirmed that the fabricated CNTFETs showed p-type characteristics in the *I*_D_–*V*_G_ curves. They applied HfO_x_, a high-*k* dielectric, as the gate dielectric for the vertical stacking of CNTFETs. Interestingly, when HfO_x_, a high-k dielectric layer, was deposited on the CNTFETs, the CNTFETs were modulated into n-type CNTFETs by electrostatic doping. They demonstrated a NOR gate as well as a vertically stacked inverter by utilizing a lower CNTFET with HfO_x_ that can be operated as n-type TFT with an upper p-type CNTFET. Furthermore, they successfully demonstrated 500 CNTFET-based vertically stacked NOR gates on a wafer scale.

In addition, several applications with vertically stacked structures using CNTs have been implemented. For example, a vertically stacked complementary inverter was fabricated by using p-type CNTFET with n-type IGZO TFT, and the integrated temperature sensor was demonstrated [102]. In addition, a CNT-based gas sensor in which the electrical properties of CNTs were modulated by gas molecules was also implemented [19]. Furthermore, the integrated electronic system based on more complicated vertically stacked structure including CNTFET was demonstrated [18], which will be discussed in the following section.

## Emerging Applications Based on Vertical Integration

### Vertical-Integrated Sensors and Optoelectronic Devices

The sensor is one of the most important functional components in a wearable electronic system, as it can actively monitor the surrounding environment and provide information to a user [103–105]. Many excellent review papers covered efforts on the development of the sensors [106–110], so we briefly introduced the vertically integrated sensors along with their strategies in this review.

**Fig. 10:**
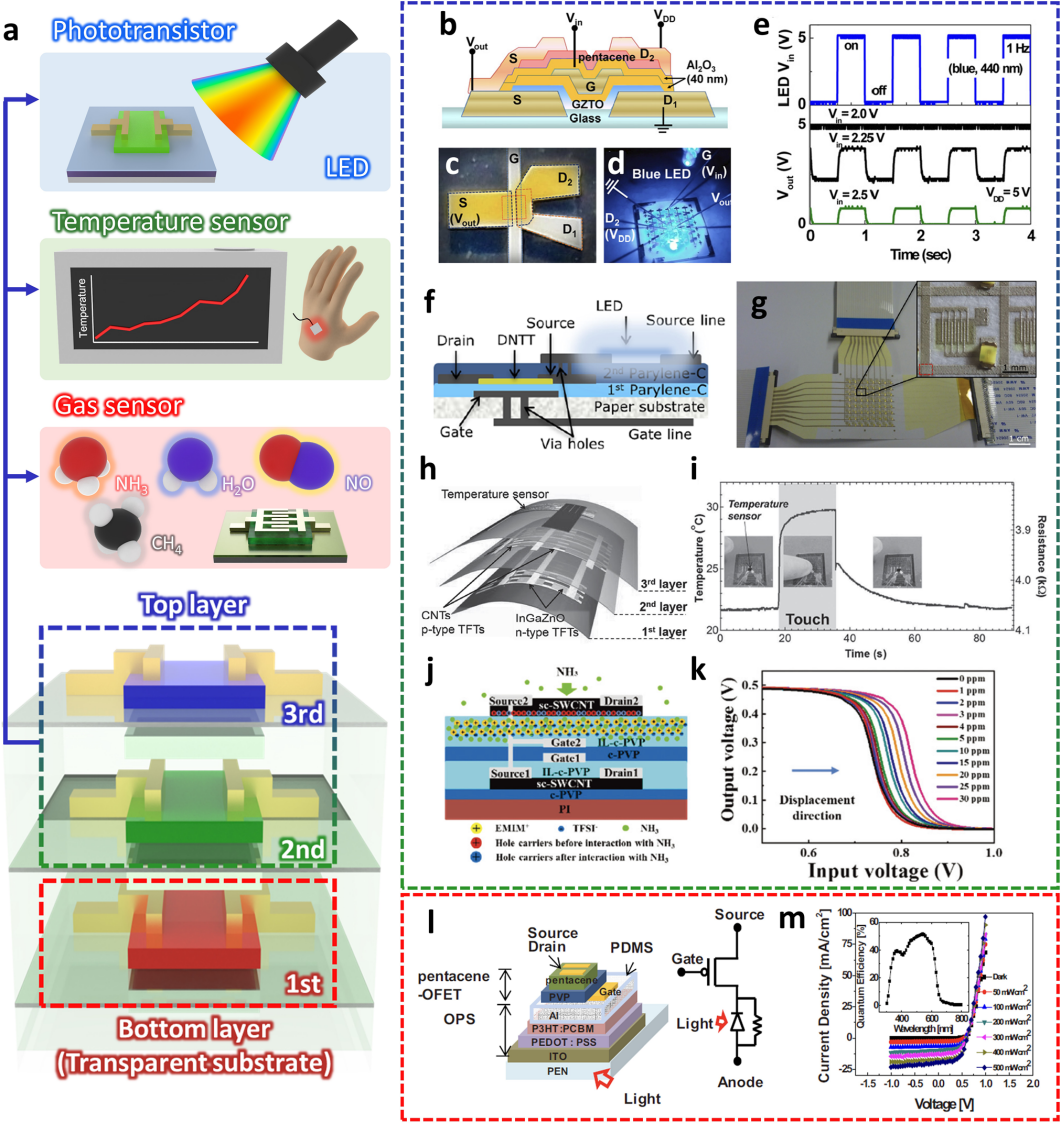
**a** Overview of top layers suitable for placing sensor elements in vertically integrated structures**. b, c** Illustration and optical microscopy image of a vertically stacked inverter with a gate-sharing structure composed of pentacene and GZTO. **d, e** Response characteristics of the vertically stacked inverter according to a blue LED pulse [27]. Copyright © 2011, Elsevier.** f** Schematic diagram of the structure that connects the LED and the driving TFT, DNTT OTFT, through a laser drill. **g** Image showing the fabricated paper-based AM LED array [22]. Copyright © 2014, Springer Nature.** h** Schematic diagram of a vertically stacked device of CNTFET and IGZO based CMOS inverter and temperature sensor on a flexible substrate.** i** Real-time temperature measurement according to human hand contact [102]. Copyright © 2015, John Wiley and Sons. **j** Illustration of an ammonia gas sensor device based on a vertically stacked SWCNT inverter. **k** Shift of output voltage curve of vertically stacked SWCNT inverter with ammonia gas concentration [19]. Copyright © 2022, RSC Publishing.** l** Vertically integrated structures of OPS and OTFT and their equivalent circuits.** m** Current density–voltage characteristic of OPS device according to light intensity and curve of measured EQE [111]. Copyright © 2010, AIP Publishing

In the vertically integrated sensor, the sensor device should be positioned to the uppermost layer and be exposed to the external environment in order to improve the sensitivity of the sensor. Therefore, various sensors such as phototransistors, temperature sensors, and gas sensors have been implemented on the top layer of the vertically integrated element (Fig. 10a). On the other hand, the position of the sensing layer is relatively free in the phototransistors and LED applications if they are fabricated on transparent substrates. In general, a transparent electrode such as ITO lessens the requirement that a photoactive or photo-generation layer resides on top of a vertical stack. In addition, in the vertical stacking of metal oxides and organic semiconductors, organic semiconductors are commonly placed on top of metal oxide semiconductors in order to prevent damage to the organic semiconductors from complex processes such as sputtering of metal oxides and consequent thermal stress. Alternatively, in the case of organic semiconductors that can be damaged by the ambient air, they are located on the bottom of the vertical stack and are encapsulated by the upper layers/devices. Including these vertical stacking designs and application rules, interesting structures of vertically stacked inverters in which various semiconductor materials are combined have been reported. Park et al. [27] demonstrated vertically stacked inverters based on pentacene and gallium zinc tin oxide (GZTO) semiconductors. They introduced a shared gate structure for manufacturing the vertically stacked inverter and placed a GZTO TFT on the bottom layer and a pentacene OTFT on the top layer (Fig. 10b, c). The low-voltage operation (< 3 V) was achieved by using Al_2_O_3_ as a gate dielectric layer, and the fabricated inverter showed full swing characteristics and obtained a DC gain up to 52 V/V. The photo-gating effect was demonstrated, by measuring the electrical characteristics of the inverter under red, green, and blue LEDs (Fig. 10d). It was found that the switching voltage of the inverter was positively shifted only under the blue LED, and the photo-gating characteristics when the pulse of the blue LED was applied were examined for the input voltage of the inverter (Fig. 10e).

Peng et al. [22] fabricated the OTFT based on the parylene dielectric layer and used it as an active-matrix (AM) array for light-emitting diode (LED). The driving dinaphtho[2,3-b:2′,3′-f] thieno[3,2-b] thiophene (DNTT) OTFT was integrated into LED by connecting the electrodes through a via-hole created by laser drilling (Fig. 10f). The bias signals were applied to AM array by using multiplexers, and the paper-based AM LED array was successfully demonstrated (Fig. 10g).

Vertical stacking of organic and oxide semiconductors-based TFTs can also be extended to integrate sensors. As an example, Honda et al. [102] implemented a temperature sensor (3rd layer) into the inverter in which IGZO TFT (1st layer) and CNT TFT (2nd layer) were vertically stacked (Fig. 10h). They introduced the polyimide (PI) layer to prevent cross-talk through isolation between each layer. In addition, the photosensitive PI enabled the formation of via-contacts for interconnection between the bottom and top layers through a photolithography process. The fabricated vertically stacked inverter showed stable operation without change in DC gain or switching voltage even after 1,000 bending cycles. They demonstrated temperature sensing through the time-varying resistance when a human finger touched the integrated temperature sensor (Fig. 10i). Additionally, the electrical characteristics of the vertically stacked inverter were measured with different temperature, which revealed that the sensitivity was − 0.0165 V °C^−1^.

Deng et al. [19] developed a single-walled carbon nanotube (SWCNT) integrated circuit on the PI substrate by printing process. The ionic liquid crosslinked PVP (IL-c-PVP) was utilized as a dielectric layer, which enabled low operating voltage of SWCNT TFT (< 1 V) and gas sensing. The electrical characteristics of the SWCNT TFTs on the bottom layer including mobility and *I*_on_/*I*_off_ were not changed after the SWCNT TFT fabrication on the top layer. There was a slight difference in the device performance of SWCNT TFTs between the top and bottom layers because the active layer of the SWCNT TFTs on the top layer was exposed to ambient air. The SWCNT TFTs and vertically integrated inverters fully maintained their electrical characteristics during the repeated bending cycles up to 10,000 times. Based on the vertically stacked SWCNT inverter, an ammonia gas sensor was demonstrated. As shown in Fig. 10j, when the device was exposed to the ammonia gas, the IL-c-PVP dielectric layer in the top SWCNT TFTs could absorb the ammonia gas. The absorbed ammonia could neutralize holes because ammonia is a strong electron donor. Consequently, the channel resistance of the top SWCNT TFTs was increased and *V*_th_ was shifted to a negative direction while the electrical characteristics of the bottom SWCNT TFTs remained unchanged, which led to the gradual shift in the VTC of the SWCNT inverter according to the ammonia concentration (Fig. 10k). Also, through the high-temperature desorption, the electrical characteristics of SWCNT TFT and inverter could be fully recovered to the initial state.

Jeong et al. [111] developed an organic photosensor (OPS) that was vertically integrated with OTFT (Fig. 10l). Poly(3,4-ethylenedioxythiophene):poly(styrenesulfonate) (PEDOT:PSS)-coated indium tin oxide (ITO) was used as anode, and poly(3-hexylethiophene)/phenyl-C61-butryic acid methyl ester) (P3HT/PCBM) was utilized as an active layer in the OPS. For the cathode, the Al electrode was used to block the light into pentacene OTFT, thus preventing the photoactivation of pentacene. Poly(dimethylsiloxane) (PDMS) with 1 mm thickness was prepared to electrically isolate the OPS from OTFT, followed by OTFT fabrication, and those devices were fabricated on a flexible PEN substrate. The anode-source current (*I*_AS_) increased when the light intensity increased in different gate bias conditions. The photoresponse increased with the increasing gate-source voltage (*V*_GS_), which showed the tunable optical properties of the integrated device according to the channel resistance of the OTFT (Fig. 10m).

### Advanced Applications Based on Vertical Integration

**Fig. 11:**
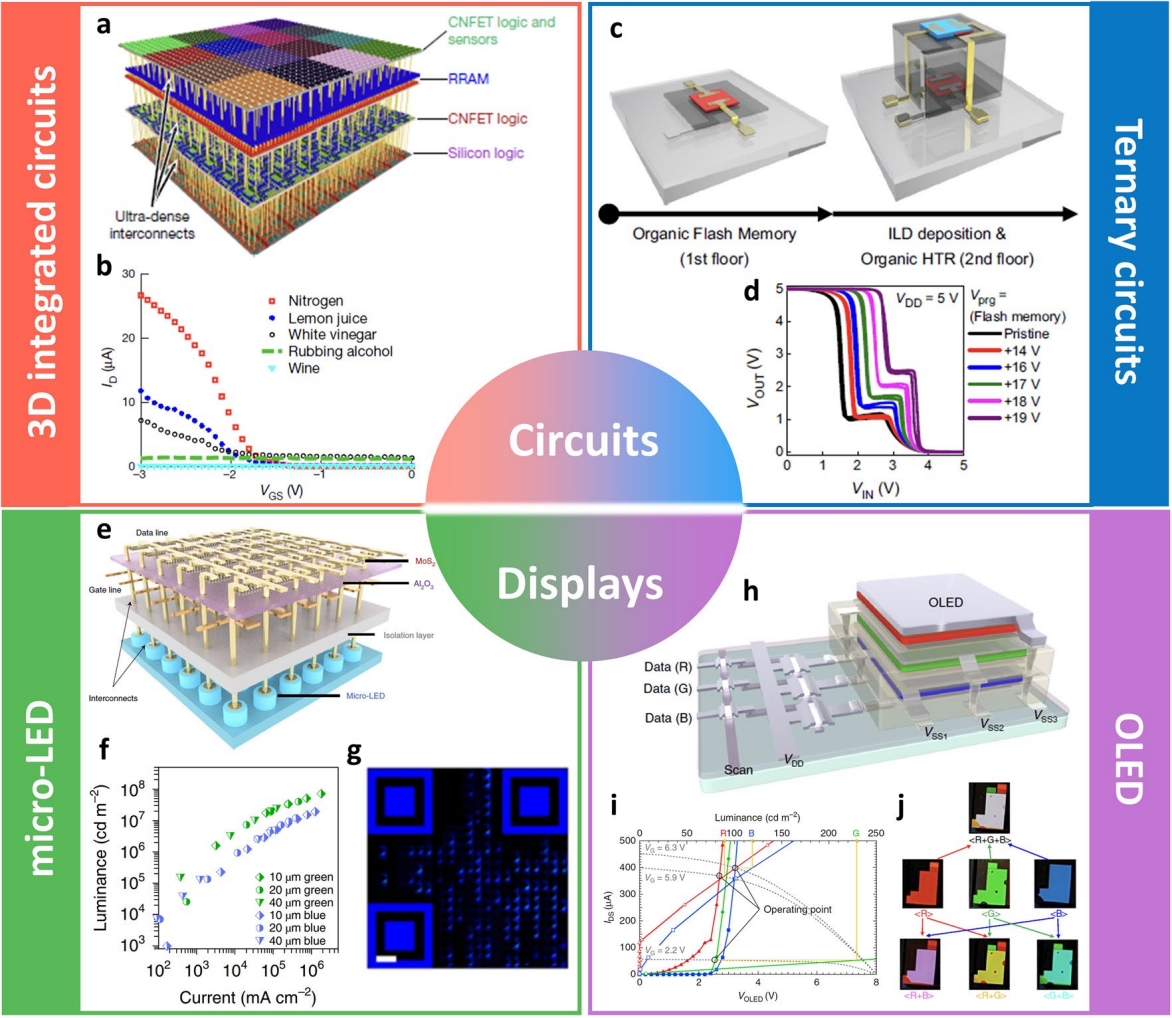
**a** Illustration of a nanosystem consisting of four stacked layers with different functions such as silicon FET logic, CNTFET logic, RRAM, CNTFET sensor, and logic. **b** Detection of various gas components by changing the electrical properties of functionalized CNTFET gas sensors [18]. Copyright © 2017, Springer Nature. **c** Organic ternary logic inverter in which flash memory and heterojunction transistors are vertically stacked in a via-hole-less metal interconnection scheme. **d** Optimization of the intermediate logic state of the ternary inverter according to flash memory state determined by the programming voltage [113]. Copyright © 2022, Springer Nature. **e** Schematic illustration of the AM micro-LED display. **f** Luminance and current of 10–40 μm blue and green micro-LEDs with a 1T1D structure normalized by the area of the micro-LED. **g** Optical microscope image of the QR code implemented with the high-resolution AM blue micro-LED display at a system level, consisting of 1,024 pixels [116]. Copyright © 2021, Springer Nature. **h** Schematic diagram of TFT-driven full-color OLED with a structure in which red (R), green (G), and blue (B) units are vertically stacked. **i** Current-luminance characteristics of vertically stacked TFT-driven full-color OLEDs in which R, G, and B pixels. **j** Multi-color realization through R, G, and B combination of TFT-driven vertically stacked full-color OLED [119]. Copyright © 2020, Springer Nature

The most important advantage that can be achieved from monolithic 3D integration through vertical stacking is the increased data processing capability in the given 2D area, as we repeatedly emphasized in this paper. Shulaker et al. [18] developed a 3D integrated circuit by combining the device technologies based on emerging materials together, which is regarded as an important milestone in 3D integrated electronics. A prototype of the functional device was demonstrated, where sensing, data storage, and computing could be processed in a single chip. The developed nanosystem consisted of 4 different layers, each of which has a different role (Fig. 11a). The silicon transistors were fabricated on the 1st layer due to the high processing temperature. Those conventional devices interfaced with other layers to read RRAM in the 3rd layer and to steer these data to a CNTFET computing system. The CNTFET-based classification accelerator on the second layer is computed on the input data acquired from the CNTFET gas sensors on the fourth layer. The third layer consisted of the non-volatile RRAM cells, which provided data storage by being integrated with the silicon select transistors. On the topmost (4th) layer, a huge number (more than one million) of CNTFET inverters were fabricated and they were operated as chemical vapor sensors. Such a complex, high-density integrated circuit was trained to distinguish shared gases and vapors including nitrogen, the vapors of lemon juice, white vinegar, rubbing alcohol, vodka, wine, and beer (Fig. 11b). Also, it is worthwhile to note that all the components could be operated within a low voltage of less than 3 V. This work showed the process compatibility of the emerging materials with current silicon-based technology, thus demonstrating a functional prototype. In addition, the logic devices were successfully integrated with memories in a single chip by using a vertically stacked structure, which can overcome the main bottleneck arising from the data transfer between off-chip memory and on-chip logic circuits.

Multi-valued logic (MVL) circuits have been spotlighted, as they can increase data processing capability by using the intermediate logic states between conventional logic states (0 and 1) For example, system complexity can be theoretically reduced to ~ 63% in a ternary logic circuit compared to the conventional binary logic circuit [112]. The intermediate logic state can be implemented by utilizing a heterojunction transistor, which enables the ternary logic devices without increasing number of devices. Choi et al. [113] developed the organic ternary logic inverter in a vertically stacked structure (Fig. 11c). The dielectric layer was patterned during the deposition, which enables metal interconnection in different layers. The iCVD process was utilized to fabricate the ultrathin polymer dielectric layers, which enabled low-voltage operation (< 5 V) as well as high uniformity in the electrical characteristics of the fabricated devices. Moreover, non-volatile flash memory was implemented and integrated with the heterojunction transistor in a vertically stacked manner. The intermediate logic state of the ternary logic circuit was systematically optimized with the appropriate programming/erasing operation of the flash memory (Fig. 11d). In addition, by designing the dielectric materials according to their dielectric constant (high-*k* dielectric for blocking dielectric layer and low-*k* dielectric for tunneling dielectric layer), low-voltage programming/erasing (< 19 V) was achieved. The vapor-phase deposited, highly robust polymer dielectric materials enabled the excellent retention characteristics of the flash memory, leading to a reliable operation of the ternary logic inverter. This study provided a useful insight to achieve high-performance MVL circuits and the information density per unit area was further improved by introducing vertically stacked structures into MVL circuits.

The most important components that consist of AM displays are TFT and LED. Each pixel is controlled by the individual TFT, which makes AM displays have advantages such as high response time and color resolution [114, 115]. There have been researching efforts to demonstrate high-density AM displays in a vertically stacked manner by exploiting the emerging materials for next-generation advanced display systems. Meng et al. [116] demonstrated large-area MoS_2_ TFTs that were vertically integrated with GaN-based micro-LED through the back-end of line (BEOL) integration (Fig. 11e). The GaN-based LED was fabricated by MOCVD in the bottom layer and the MoS_2_ TFT array lay on top of the micro-LED. An ultraclean process for MoS_2_ TFTs was developed, which enabled high mobility (~ 54 cm^2^ V^−1^ s^−1^). Combined with the short channel length (~ 1 μm), the on-current (*I*_on_) reached 210 μA μm^−1^, which is capable of operating micro-LEDs. The device yield was as high as 95% even with a large number of the fabricated devices (~ 200 TFTs) owing to the scalable fabrication process. Through the monolithic integration, a one-transistor–one-diode (1T1D) scheme was realized, which showed extremely high brightness (luminance of 7.1 × 10^7^ cd m^−2^) (Fig. 11f). Moreover, a quick response (QR) image was demonstrated as an example of the high-resolution AM display at a system level, consisting of 1024 pixels with 20 μm pitch, which corresponds to 1270 pixels per inch (PPI) (Fig. 11g). This work shows the compatibility of atomically thin semiconductors with the existing display technologies and their potential for advanced display applications.

Organic light-emitting diodes (OLEDs) have been positioned as a mainstream of displays including mobile devices and TVs owing to their high efficiency, light-weight, and high-color gamut [117, 118]. To meet the requirements for future display systems such as augmented reality (AR) and virtual reality (VR), a high-resolution display is highly recommended. Choi et al. [119] developed a vertically stacked OLED system by using intermediate electrodes. The transparent indium zinc oxide (IZO) intermediate electrodes were patterned by the photolithographic process to achieve a finely patterned, high-resolution display. The damage on the OLED devices potentially caused during the photolithography process was prevented by the additional SiN_x_ passivation layers on top of the Al_2_O_3_ thin-film encapsulation (TFE), where the insufficient protection of Al_2_O_3_ TFE was supplemented by transparent, low-temperature processed SiN_x_ passivation layer. The device structure including the thickness of each layer was optimized by the optical simulation to ensure high efficiency and color gamut. Based on the optimized structure, the independent control of red (*R*), green (*G*), and blue (*B*) units was achieved as well as low-operating voltage (turn-on voltage lower than 2.6 V) and sufficient luminance (up to 930 cd m^−2^) in the fabricated vertically stacked OLEDs. Finally, TFT-driven full-color OLED was demonstrated based on the vertically stacked structure (Fig. 11h). Al-doped In–Zn–Sn–O (IZSO) driving TFTs were fabricated before the OLED deposition due to their high processing temperature, and they showed the low *V*_th_ of 0.29 V as well as high saturation mobility of 16.3 cm^2^ V^−1^ s^−1^, which resulted in adequate on-current characteristics. By adopting two transistor-one capacitor (2T-1C) pixel structures, TFT-driven individual *R*, *G,* and *B* units were successfully demonstrated, yielding 90, 230, and 120 cd m^−2^ of luminance, respectively (Fig. 11i). Various colors could be expressed by combining individual colors (Fig. 11j). By developing the photolithography-processed fine pattern of the intermediate electrodes, they demonstrated the vertically stacked, full-color OLED driven by TFT for the first time, which showed the great potential of OLED for a high-resolution display system.

**Fig. 12:**
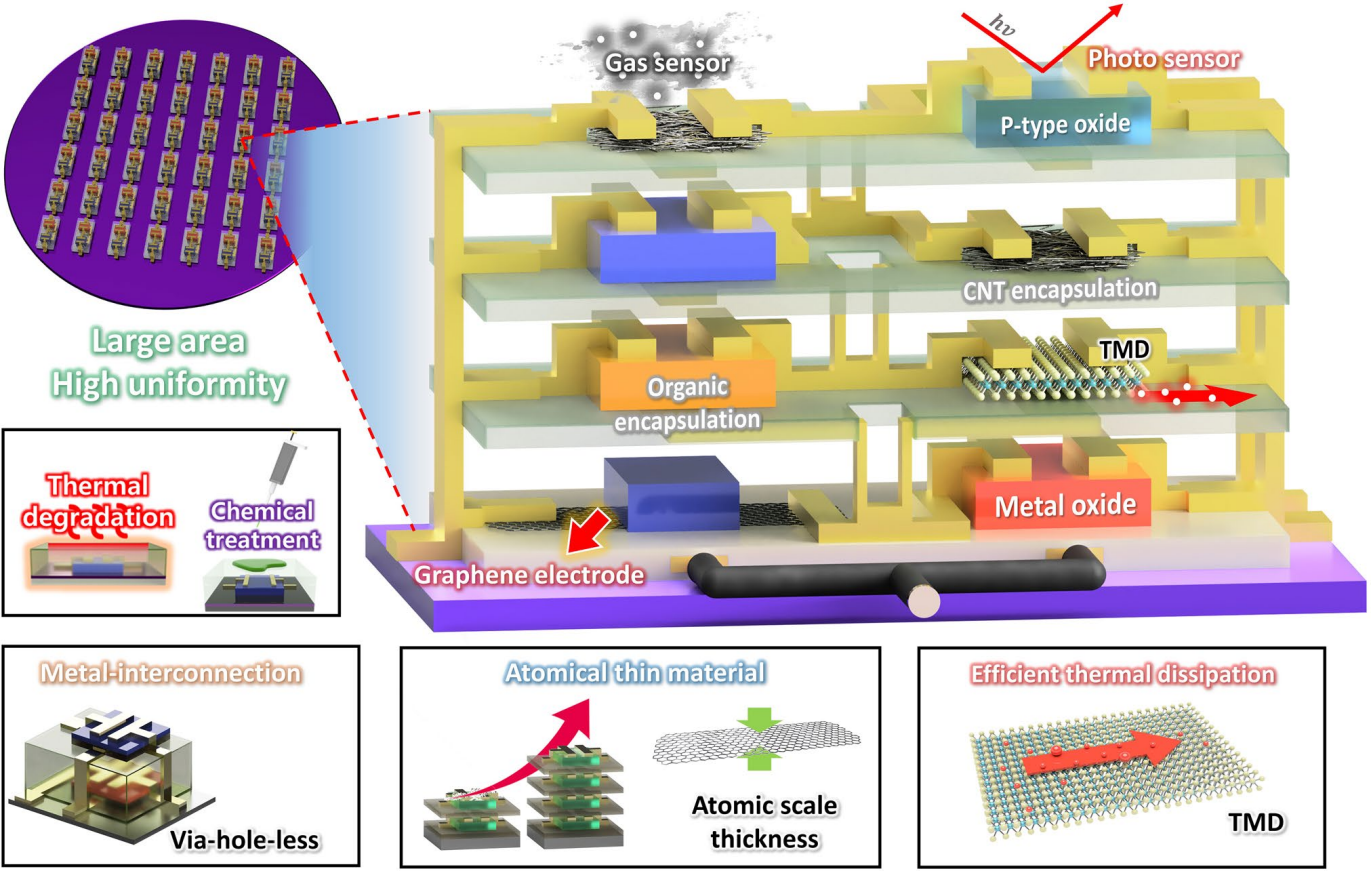
An overview of 3D integration based on reliable metal interconnections and future applications of various semiconductor materials

**Table 1:** Summary of previously reported emerging material-based vertical stacking applications

1st layer	2nd layer (3rd layer or more)	Dielectric	Layer (#)	Structure		Inter-connection	Application	Operating voltage (V)	References
*Organic semiconductor-based vertical stacked devices*									
Pentacene	Pentacene	PMMA	2	Separated devices		–	–	− 80	[23]
Pentacene	Pentacene	PMMA, PVPh	2	Single gate		Shadow mask pattern	Inverter	− 20	[57]
TIPS-pentacene/PTAA	PCBM	CYTOP/Al_2_O_3_, Al_2_O_3_	2	Shared gate		–	Inverter	8	[20]
DNTT	LED	Parylene	2	Active matrix		Via-hole (Laser drilling)	Active-matrix driver	− 80	[22]
P(NDI2OD-T2)	TIPS-pentacene	CPVP, CYTOP/CPVP	2	Shared gate		Externally connection	Inverter, NOR, NAND	30	[72]
TU-3	diF-TES-ADT/PS	Parylene	2	Shared gate		Via-hole (Laser drilling)	Ring oscillator	1	[67]
P-24-PNDI-TVT	P-29-DPP-SVS	CYTOP/CPVP, PMMA	2	Shared gate		–	Inverter	30	[120]
PTCDI-C13	PTCDI-C13 (DNTT, DNTT, DNTT)	pV3D3	5	5 separated gates		Via-hole-less (Dielectric patterning)	Inverter, NOR, NAND	8	[39]
TU-3-PαMS	TU-3-PαMS (C8-BTBT-PαMS)	Parylene	3	Single gate, shared gate		Via-hole (Laser drilling)	SRAM	3	[121]
*Oxide semiconductor-based vertical stacked devices*									
CuO	IGZO	Al_2_O_3_	2		Shared gate	–	Inverter	10	[24]
SnO	IGZO	Al_2_O_3_	2		Shared gate	Via-hole (Wet etching)	Inverter, photo-sensor	6	[25]
*Organic oxide-based vertical stacked devices*									
F8T2	IGZO	–	2		Shared gate	–	Inverter	10	[90]
GZTO	Pentacene	Al_2_O_3_, P(VDF-TrFE)	2		Shared gate	Wet etching	Inverter, photo-sensor, memory	3	[27]
IGZO	Pentacene	Al_2_O_3_	2		Shared gate	–	Inverter	4	[91]
ZnO	TIPS-pentacene	Silicone resin	2		Shared gate	Via-hole (Photolithography)	Inverter, NAND, NOR	10	[26]
*CNT-based vertical stacked devices*									
IGZO	CNT (CNT-PEDOT:PSS)	Al_2_O_3_/SiO_x_	3		2 separated gates	Via-hole (Wet etching)	Inverter, temperature sensor	5	[102]
CNT	CNT	HfO_x_	2		Shared gate	–	Inverter	1	[17]
CNT	CNT	IL-c-PVP	2		2 separated gates	Via-hole (Laser drilling)	Inverter, gas sensor	0.3	[19]
*2D materials-based vertical stacked devices*									
Bi_2_Sr_2_Co_2_O_8_	MoS_2_	SiN_x_	2		Vertical transistor	–	Inverter	− 2	[97]
MoS_2_	MoS_2_	SiO_2_	2		Global back gate	–	–	–	[95]
MoS_2_	WSe_2_	ZrO_2_	2		Shared gate	-	Inverter, NAND, NOR, amplifier, mixer	0.5	[98]
Si nanowire	MoS_2_	–	2		–	–	Phototransistor	40	[99]
Si	Graphene (PbS QDs)	–	3		Photoconductor	–	Image sensor array	–	[16]
MoS_2_	WSe_2_	ZrO_2_	2		Shared gate	–	Inverter	0.5	[12]
MoS_2_	MoS_2_	Al_2_O_3_/HfO_2_	2		Gate-all-around	–	–	–	[96]
MoS_2_	MoS_2_ (MoS_2_)	hBN	3		Gate-all-around	–	Memory, inverter, NAND, phototransistor	1	[13]
Te	Te	ZrO_2_	3		Single gate	Via-hole (Wet etching)	Inverter	2	[32]
MoS_2_	MoS_2_ or WSe_2_	HfO_2_	2		Gate-all-around, shared gate	–	Inverter	3	[14]

## Conclusion and Outlook

In summary, we reviewed recent progress in monolithic 3D integration of electronic devices (Fig. 12 and Table 1). Numerous research efforts have been dedicated to achieving vertical integration by exploiting emerging semiconductor materials including TMDs, organics, metal oxides, and CNTs. Also, bottom-up processes that can be suitable for emerging semiconductor materials have been established. The primary benefit that can be achieved from vertical integration is increased device density. The number of transistor per given area can be enhanced in vertical integration, and the integration density can be further increased as the circuit become complex where the required number of the transistor is increased. In addition, by placing the transistor with the ambient-instable semiconductors such as n-type organic materials and some 2D semiconductors on the bottom layer, the air stability of the device can be improved. Furthermore, compared to lateral structure, it is relatively easy to optimize the dielectric interface and charge injection for each semiconductor material. In other words, dielectric materials and their thickness, and work function of *S*/*D* electrodes can readily be adjusted in vertical structure, to improve the device performance. In addition to the logic circuits, vertical integration of transistors with other functional devices including sensors, memories, and light-emitting diodes has been recently demonstrated to develop advanced sensors, circuits, and display systems, as we revisited in this review. However, there are still challenges that need to be resolved as follows:

(i) Heat dissipation and power consumption should be considered. With the increasing number of the transistor devices per unit area, more heat can be generated. Moreover, in the vertically stacked structure, heat is hard to dissipate, since the device on the bottom layer is buried in the insulating films. Therefore, it is highly demanded to develop materials and architectures for heat sink that can properly release heat generation from the vertically integrated devices. The power consumption is another important factor that should be taken into account, as the integration density is increased. Since the dielectric capacitance determines operating voltage of the unit transistor, it is important to reduce the thickness of the insulating layer. However, the dielectric layers fabricated via bottom-up processes (deposition processes) typically showed the limited insulating performance compared to the standard thermally grown silicon dioxide. Furthermore, mechanically flexible insulating films such as polymers typically show poor insulating performance compared to the inorganic materials when the thickness is reduced. The use of high-*k* dielectric materials is alternative way to achieve low-voltage operation; however, potential side effects including charge scattering and trap generation at the semiconductor/dielectric interface should be considered. In addition, appropriate circuit design should be accompanied to reduce power consumption, as in the lateral device structure.
(ii) High uniformity and device yield should be secured. Most bottom-up processes for transistor devices based on emerging semiconductors require thermal treatment to improve the film quality and electrical characteristics of each layer. The deposition processes can also induce thermal stress on the underlying layers and devices. In the vertically stacked structure, thermal stress can be accumulated with the increasing number of integration, which may cause degradation in the underlying devices. Therefore, it is critically important to optimize the process conditions that can minimize the change in the electrical characteristics of the underlying devices, to ensure uniformity and yield in the vertical direction. The via-hole forming process to make electrical contact between metals in different layers, is another sensitive procedure. Laser drilling and soft etching by organic solvents have been suggested to remove the organic layers in a selective area, and wet etching has been widely utilized for patterning the inorganic layers. However, such destructive methods may cause the damage to the underlying devices and substrates, because the semiconductor materials and flexible substrates are vulnerable to thermal energy or chemicals. Therefore, considerable efforts are highly demanded to develop reliable methods to selectively remove or pattern dielectric layers according to the material properties.
(iii) Device performance and pattern resolution should be improved. It is worthwhile to discuss the device performance and pattern resolution, even though these are also highly required in the lateral devices. The high charge mobility and low bulk/interface trap density, as well as mechanical deformability, are important in the next-generation electronics. With the huge research efforts in last two decades, electrical and mechanical properties of organic semiconductors have been improved. However, their electrical characteristics are still far from satisfaction, compared to the silicon devices. Metal oxide semiconductors typically exhibit high charge mobility; however, their mechanical flexibility and operational stability need to be improved. Also, discovering high-performance p-type metal oxide semiconductors is still demanded. Atomically thin 2D materials including TMDs are emerging semiconductors because of their unique electrical properties. Nevertheless, current 2D semiconductor devices rely on mechanical exfoliation and large-area synthesis methods require high process temperature. Therefore, appropriate processes should be established to utilize the excellent electrical properties of 2D semiconductor materials for practical use. Reducing channel length is another way to obtain a large amount of current. However, conventional photolithography-based patterning may not be directly applied to some emerging semiconductors due to their limited thermal and environmental stability; thus, developing an alternative way to achieve short channel devices is required. In addition, patterning dielectric layers are important to reduce overall dimension, as well as to make a metal interconnection between different layers.

In the vertically stacked structure, there are big differences in material selection and process design, compared to conventional lateral device geometry. In spite of the challenges discussed above, vertical integration has been spotlighted, because this approach can enable us to circumvent the scaling limitation that current silicon technology encounters. Therefore, huge research efforts are still desperate to maximize the advantages of vertical integration. We believe the vertical 3D integration based on emerging semiconductors is an attractive strategy to accommodate high demand of data processing in future wearable electronics and Internet-of-Things (IoT).
